# Protective Effect of *Dictyophora rubrovolvata* Extract on Intestinal and Liver Tissue Toxicity Induced by Metformin Disinfection Byproducts

**DOI:** 10.3390/toxics13040310

**Published:** 2025-04-16

**Authors:** Huijuan Liu, Dong Xiang, Jie Zhou, Jiao Xie

**Affiliations:** 1 Key Laboratory of Environmental Pollution Monitoring and Disease Control, Ministry of Education, Department of Nutrition and Food Hygiene, School of Public Health, Guizhou Medical University, Guiyang 561113, China; 18744851710@163.com; 2 Guangdong Provincial Key Laboratory of Malignant Tumor Epigenetics and Gene Regulation, Guangdong-Hong Kong Joint Laboratory for RNA Medicine, Sun Yat-Sen Memorial Hospital, Sun Yat-Sen University, Guangzhou 510120, China; zhouj288@mail2.sysu.edu.cn

**Keywords:** *Dictyophora rubrovolvata* extract, natural product, metformin disinfection byproducts, organ toxicity, gut microbiota, gut-liver axis

## Abstract

Metformin disinfection byproducts Y and C have emerged as pollutants of concern in drinking water systems and are suspected to possess significant toxicity to mammals. However, effective strategies to mitigate the effects of Y and C exposure in mammals have not been thoroughly formulated. This study aimed to investigate the toxicity and characteristic phenotypes of short-term, high-dose exposure to Y and C in the intestine and liver of mice and to evaluate the protective effects of *Dictyophora rubrovolvata* extract (DRE) on Y- and C-induced intestinal and liver damage. The results showed that exposure-induced intestinal toxicity manifested mainly as intestinal barrier dysfunction, induction of immune response and oxidative stress, and disruption of intestinal flora homeostasis. Hepatotoxicity was mainly characterized by histopathological changes such as vacuolar degeneration, abnormal liver function, and oxidative stress. Additionally, marked changes in gut microbiota and biochemical indicators were closely related to hepatic and intestinal injuries after exposure. DRE effectively alleviated Y- and C-induced intestinal and liver damage, reshaped the gut microbiota, and maintained gut–liver axis homeostasis. These findings provide new insights into the toxic effects of disinfection byproduct exposure through the gut-liver axis and suggest that functional food extracts may serve to protect against these adverse health outcomes.

## 1. Introduction

Metformin (MET) is a biguanide antidiabetic drug that has been used clinically for many years as a first-line treatment for type 2 diabetes. In recent decades, MET has also been found to have a wide range of effects beyond hypoglycaemia, and its benefits in various diseases, including cancer, obesity, aging, and liver, cardiovascular, neurodegenerative, and kidney diseases, have been widely demonstrated [1,2,3,4,5,6,7]. Although the beneficial effects of MET continue to be discovered and its mechanism of action are being revealed, the hidden risks cannot be ignored. Owing to its widespread use, extensive discharge, and incomplete removal, MET has continuously accumulated in aquatic environments, and as a typical emerging contaminant, it can be converted into a variety of byproducts during sewage treatment and drinking water disinfection processes that pose a potential threat to ecosystems and human health [8,9,10,11]. Indirect or direct discharges of industrial and medical wastewater may be important sources and pathways through which MET and its byproducts enter aquatic environments [12]. Recently, MET and its byproducts found in aquatic environments have been shown to induce abnormal physiological processes, endocrine disorders, oxidative stress, intestinal damage, and reproduction abnormalities in aquatic organisms; for example, the LC50 of MET for *Oryzias latipes* embryos reached 383.3 mg/L, and the transcriptionally upregulated VTG gene induced estrogenic effects in males of the exposed group [13]. In fact, MET and its byproducts are frequently detected in water systems, including drinking water systems, worldwide [9]. Therefore, it is necessary to study the possible effects of MET and its byproducts on the sustainable supply of safe drinking water and human health.

Owing to its simplicity of application, low cost, and efficient bactericidal effects, chlorination is a widely used drinking water disinfection method worldwide [14]. Research has shown that active chlorine can oxidize MET to generate two emerging nitrogen-containing disinfection byproducts (DBPs), Y (C_4_H_6_ClN_5_) and C (C_4_H_6_ClN_3_), during disinfection processes in urban water plants [15,16]. In the presence of the same concentration of chlorine, the production of these two byproducts gradually increases with increasing MET concentration [16]. Additionally, simulated disinfection and stability analyses have strongly confirmed that both byproducts form rapidly and remain stable under certain conditions, implying that the users of the treated water have a high probability of being exposed to these byproducts [16]. The prevalence and production of these two chlorination byproducts have been investigated in urban water supply systems in China, and their widespread presence and possibility of exposure have been confirmed [9]. A 2019 survey of metformin and C concentrations in tap water across 38 Chinese cities confirmed that the distribution patterns of metformin and its disinfection byproducts were similar to those of antibiotics and other pharmaceuticals in China, highlighting the significant role of anthropogenic factors in the contamination. Among the surveyed cities, Shanghai showed the highest detected concentration of metformin (336.7 ± 11.4 ng/L), Suzhou had the highest concentration of C (9.72 ± 0.38 ng/L), and the detection status of Y was limited by analytical methodology [9]. Thus, if MET is continuously released and accumulated in drinking water systems, the threats of these two chlorination byproducts to the health of the water users will also increase. Recent studies have confirmed the toxic effects of these two chlorinated byproducts to *Escherichia coli*, *Pseudokirchneriella subcapitata*, the liver cancer cell line HepG2, *Caenorhabditis elegans*, and mice. These toxic effects have manifested as the destruction of cell structure, composition and function, the accumulation of reactive oxygen species (ROS), and the induction of oxidative stress [9,16,17]. In addition, Y, with a triazole-derived structure, and C, with a chlorinated organonitrile structure, are highly nitrogenous substances. Their unique chemical structures make them more toxic to nematodes and HepG2 cells than arsenic [16]. Increasing evidence highlights the importance of intestinal homeostasis, which is closely related to intestinal structural integrity, intestinal immunity, intestinal oxidative stress, the intestinal microflora, and the interactions among these factors, in health maintenance [18]. The disruption of intestinal homeostasis by harmful exogenous factors, such as disinfection byproducts, leads to intestinal dysfunction [19]. In addition to the intestine, the liver is another important target organ affected by harmful exogenous factors. Moreover, hepatotoxicity caused by harmful exogenous factors may be amplified in individuals with disrupted intestinal homeostasis [18]. However, the manifestations of Y and C toxicity to the gut-liver axis remain to be further elucidated.

Owing to the high risk of exposure to, and complex biological toxicity of, drinking water disinfection byproducts [20], it is imperative to develop practical strategies to prevent the ensuing health hazards. *Dictyophora rubrovolvata* is a well-known edible and medicinal fungus. Its extract is rich in polysaccharides, polyphenols, and other bioactive components and has shown immunoregulatory, antioxidant, and other physiological effects [21]. In an experimental arsenic-induced liver injury model, *Dictyophora rubrovolvata* extract (DRE) counteracted arsenic-induced hepatic stellate cell activation and liver fibrosis by regulating upstream and downstream proteins in the MAPK pathway [22]. Moreover, the extent of the liver tissue lesions and liver function abnormalities caused by carbon tetrachloride (CCl_4_) can be greatly alleviated by DRE [23]. Importantly, DRE can regulate the inflammatory response and oxidative stress to reduce the degree of intestinal damage in dextran sodium sulfate (DSS)-induced ulcerative colitis [24]. Thus, we hypothesized that DRE could alleviate Y- and C-induced intestinal and liver damage by modulating the tissue redox status and immune homeostasis.

The adverse effects of disinfection byproducts in drinking water on the intestines and liver have been investigated in previous studies, but they did not compare the extent of the damage to various organs. Therefore, in this study, the effects of Y and C on two important target organs, the intestines and liver, were examined under the identical exposure conditions. Herein, we exposed mice to the MET chlorination byproducts Y and C, conducted histopathological and biochemical examinations to evaluate intestinal and liver damage, and analyzed the intestinal microbiome in the cecal contents using 16S RNA sequencing. The exposure groups receiving DRE treatment were employed to evaluate the protective effects of DRE. These data may provide important information for the further exploration of the long-term health effects of exposure to disinfection byproducts and support the development of practical prevention strategies.

## 2. Materials and Methods

### 2.1. Chemicals and Reagents

Fresh fruiting bodies of *D. rubrovolvata* were collected from Baiyun District, Guizhou Province, China. MET was purchased from Wuhan Jingbiao Technology Co., Ltd. (Wuhan, China). Sodium hypochlorite solution (14% active chlorine basis), dichloromethane, and ethyl acetate, all of analytical grade, were obtained from Aladdin (Shanghai, China). Silica gel 60 (70–230 mesh) was purchased from Chongqing Platinum Strontium Titanium Technology Co., Ltd. (Chongqing, China). The catalase (CAT), superoxide dismutase (SOD), glutathione peroxidase (GSH-Px), malondialdehyde (MDA), ROS, and glutathione/oxidized glutathione (GSH/GSSG) assay kits were obtained from Nanjing Jiancheng Bioengineering Institute (Nanjing, China). Mouse interleukin 6 (IL-6), interleukin 10 (IL-10), tumor necrosis factor α (TNF-α), and interferon γ (IFN-γ) enzyme-linked immunosorbent assay (ELISA) kits, universal tissue fixative, and phosphate-buffered saline (PBS; pH 7.4) were purchased from Wuhan Servicebio Technology Co., Ltd. (Wuhan, China).

### 2.2. Preparation and Characterization of Y and C

The chlorination byproducts Y and C were prepared according to the protocol by Armbruster et al. and Zhang et al. [15,16]. Notably, in addition to the steps in the conversion process, all other steps in the compound preparation process should be performed under light- and heat-free conditions to ensure the purity of the products. Dichloromethane (for extraction) and the chromatography column filled with silica gel 60 and pre-equilibrated with ethyl acetate were precooled to 4 °C. Thin-layer chromatography (TLC) was used to facilitate sample collection after chromatography, and nuclear magnetic resonance (NMR) spectrometry was used to analyze the purities of the prepared compounds Y and C [9,15]. In addition, ultraviolet–visible (UV–Vis) absorption spectroscopy and Fourier transform infrared (FTIR) spectroscopy were also used to characterize and identify the prepared byproducts. The prepared highly pure Y (93.10%) and C (99.09%) were used for subsequent animal experiments. Notably, the purity of Y was lower than that of C because of the interconversion between these two compounds, which was consistent with previous studies [9,15,16], and this is reflected in their characterization (Figures S1–S3).

### 2.3. Preparation of the D. rubrovolvata Water Extract

The *D. rubrovolvata* water extract was prepared according to the optimal drying method, extraction conditions, and antioxidant capacity of *D. rubrovolvata*, as described by Liu et al. [21]. Specifically, the fresh fruiting bodies of *D. rubrovolvata* were dried under vacuum at 45 °C for 11 h, and then the dried samples were crushed and sieved through a 5 mm mesh. The powder samples were ultrasonically extracted for 20 min (60 °C, 20 kHz and 600 W), with a solid–liquid ratio of 1:20. For the extract, the total polyphenol content and total polysaccharide content were 2.67 ± 0.08 mg GAE/g and 0.30 ± 0.01 g/g, respectively. The DPPH radical scavenging rate, hydroxyl radical scavenging rate, and ferrous ion chelating rate were 63.44 ± 1.92%, 84.30 ± 0.15%, and 74.55 ± 2.03%, respectively [21]. After extraction, the supernatant was collected, diluted to 10 mg/mL, and stored at 4 °C for later use.

### 2.4. Animals and Experimental Design

**Figure 1:**
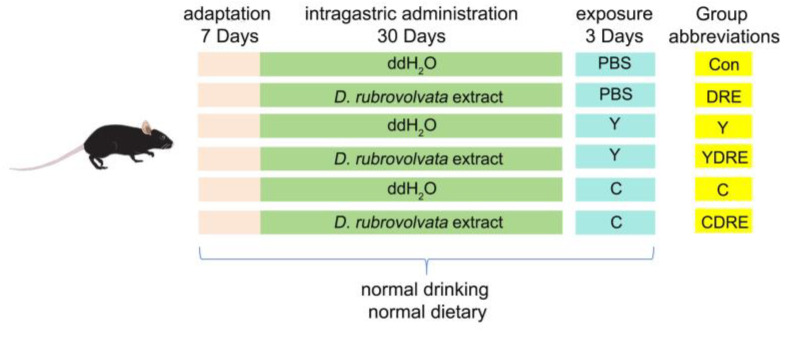
Exposure strategy used in this work.

Wild-type male C57BL6 mice, 8 weeks old (22–24 g), were purchased from the Experimental Animal Center of Guizhou Medical University (Guiyang, China). All of the experiments involving animals were approved by the Experimental Animal Ethics Committee of Guizhou Medical University (no.: 2200401) and conducted according to the guidelines of the Institutional Animal Care and Use Committee. The detailed exposure plan was depicted in Figure 1. All experimental animals were placed in presterilized cages and housed in a regularly ventilated environment with an ambient temperature of 22 ± 2 °C and a relative humidity of 40–60%. After one week of adaptive feeding, the mice were randomly divided into six groups—control (Con), DRE intervention (DRE), Y exposure (Y), Y exposure and DRE intervention (YDRE), C exposure (C), and C exposure and DRE intervention (CDRE) groups—with six mice in each group. Importantly, the average mouse weights of each group were similar at the initial stage of the experiment. The DRE, YDRE, and CDRE groups were administered with *D. rubrovolvata* fruiting body water extracted intragastrically for 30 days at a dosage of 56 mg/kg/d, whereas the Con, Y, and C groups were intragastrically administered with ddH_2_O of the same volume. One hour after the last intragastric administration, compounds Y and C were dissolved in PBS and intraperitoneally injected into the appropriate mice once a day for 3 days. The Y and YDRE groups were given Y at a dosage of 2 mg/kg/d, whereas the C and CDRE groups were given C at a dosage of 10 mg/kg/d. The Con and DRE groups were administered with the same volume of PBS. The intervention and exposure dosages were determined according to the methods of Zhang et al. and Zhang [16,25] and our preliminary experimental results (Figures S4–S6). Mice were anesthetized by intraperitoneal injection of 2% pentobarbital sodium (50 mg/kg) after being fasted for 12 h. Blood was collected from the retro-orbital plexus and mice were euthanized via cervical dislocation. Finally, the serum, cecal contents, and small intestine, colon, and liver tissues were collected for subsequent analysis.

### 2.5. Histological Analysis

The liver, small intestine, and colon tissue samples taken from the same anatomical position of each group of mice were fixed with a 4% paraformaldehyde solution and then dehydrated with alcohol at different concentrations. After paraffin-embedding and slicing, the small intestinal and colon tissues were subjected to hematoxylin and eosin (H&E) and alcian blue-periodic acid Schiff (AB-PAS) staining. The H&E staining procedure for the liver slices was the same as that used for the intestinal tissues. Image of the slices were acquired with a microscope (Nikon Eclipse E100, Nikon, Tokyo, Japan) and analyzed with an imaging system (Nikon DS-U3, Nikon, Tokyo, Japan). The histopathological scores and criteria were determined via the method described by Ye et al. and Mohammadian et al. [26,27]. According to the extent of inflammation, inflammatory cell infiltration, degree of damage to the crypts, crypt abscess, submucosal edema, loss of goblet cells, degree of crypt epithelial hyperplasia (for intestine), sinusoidal dilatation, inflammatory cell infiltration, congestion, degeneration, and cytoplasmic vacuolization (for liver) were scored as normal (0), mild (1), moderate (2), severe (3), and extremely severe (4).

### 2.6. Serum Biochemical Indices

The obtained blood samples were allowed to stand at room temperature for 2–3 h and then centrifuged at 4 °C and 3000 rpm for 15 min. The collected supernatants were temporarily stored at −80 °C. For analysis, the frozen serum samples were first thawed on ice. Then, the activities of alanine aminotransferase (ALT) and aspartate aminotransferase (AST), which are critical biochemical parameters reflecting liver function, in each group of samples were determined using an automatic biochemical analyser (Chemray 240, Shenzhen, China).

### 2.7. Determination of Inflammatory Factor Contents

The liver, small intestine, and colon tissue samples were completely homogenized in precooled PBS (pH 7.4) in an ice water bath. The concentrations of IL-6, IL-10, TNF-α, and IFN-γ in the supernatants obtained by centrifugation at 3000 rpm and 4 °C for 15 min were determined using mouse ELISA kits.

### 2.8. Evaluation of Oxidative Stress Levels

The liver and intestinal tissue samples that had been frozen at −80 °C were thawed on ice and then used to prepare tissue homogenates by adding 9 times their weight of precooled normal saline (tissue weight (g)–buffer volume (mL) = 1:9) at 4 °C. After centrifugation at 4 °C and 3000 rpm for 15 min, the supernatants were analyzed for the contents of CAT, SOD, GSH-Px, and MDA, the ratio of GSH/GSSG, and the level of ROS strictly according to the instructions of the kits.

### 2.9. Gut Microbiome Analysis

The collected cecal contents were frozen in liquid nitrogen and stored at −80 °C. DNA was extracted and purified with a Fast DNA SPIN Kit (MP Biomedicals, Thomas Irvine, CA, USA). The concentration and quality of the obtained genomic DNA were evaluated by a NanoDrop ND-2000 (Thermo, Waltham, MA, USA) after preliminary determination via 1% agarose gel electrophoresis. The specific primers (with barcodes) were synthesized according to the particular sequencing region, and then the V3–V4 variable region of 16S rRNA was effectively amplified. Then, 2% agarose gel electrophoresis was carried out to separate the amplified products, and the AxyPrep DNA Gel Recovery kit (Axygen, Union City, CA, USA) and the QuantiFluor^TM^-ST Blue fluorescence quantitative system (Promega, Madison, WI, USA) were used to recover, detect, and quantify of the PCR products. The TruSeqTM DNA Sample Prep kit (Illumina, San Diego, CA, USA) was used to construct DNA libraries by adding the Illumina official adaptor sequence to the outer end of the target region, followed by product recovery and quantification. Sequencing was carried out with a PE300 instrument on a NovaSeq 6000 platform (Illumina, San Diego, CA, USA). The DADA2 pipeline within QIIME2 was selected for noise reduction to filter low-quality and chimeric sequences and obtain unique amplicon sequence variants (ASVs). The microbiome composition of each group of samples was analyzed at multiple taxonomic levels, including the phylum and genus levels. A Venn diagram was constructed to illustrate the common or specific ASVs in each group. The distance between samples within and among the groups was evaluated via the Bray–Curtis method. β diversity was evaluated by nonmetric multidimensional scaling (NMDS) analysis, principal component analysis (PCA), and principal coordinate analysis (PCoA), using analysis of similarity (ANOSIM) as the intergroup difference test method. This series of analyses conducted on the sequencing data were carried out using the Meiji Biological One-stop Scientific Research Service platform.

### 2.10. Integrated Biomarker Response Index (IBR), Correlation Analysis, and Data Treatment

In order to understand the overall reaction of organs, the data of the biological marker were estimated with the integrated biomarker response index (IBR) [28]. In this research, representative biomarkers of histopathology, histochemistry, inflammation, and oxidative damage were selected. The detailed method of IBR calculation referred to Sun et al.’s research [28]; briefly, data were standardized to allow for direct visual comparison of the biomarker responses at the test concentrations. The mean value of each biomarker for each treatment and the mean value and standard deviation for all treatments were expressed as x, X, and SD, respectively. The standardized value (Y) for each treatment was obtained by the formula Y = (x − X)/SD. Z = Y or Z = −Y, positive or negative, depended on the activation or inhibition of the biomarker in response to exposure. The minimum value (min) for each biomarker was obtained from the standardized data (Y), and the score (S) was computed as S = Y + |min|, where S ≥ 0 and |min| was the absolute value. The radar diagram was plotted according to S value, and the S values reflected the strength of the biomarkers in response to exogenous exposure; the greater the S value, the stronger the biological effects. The R_IB_ corresponded to the total area displayed by the radar diagram, and larger area indicated stronger biomarker responses and a more serious impact of chemicals on the organism. The correlation analysis of biomarkers and intestinal microbiota in each treatment group was carried out by the Spearman correlation coefficient.

Data processing, statistical analysis, and data visualization were completed with both IBM SPSS 25 (SPSS Inc., Chicago, IL, USA) and GraphPad Prism 8 (GraphPad Software Inc., La Jolla, CA, USA). One-way ANOVA and Tukey’s post hoc multiple comparisons test were used for comparisons between groups. All data were presented as the means ± standard deviations (SDs). The normality of the variables was confirmed by the Shapiro–Wilk test, and the homogeneity of variance by the Levene’s test. A non-parametric Kruskal–Wallis test was used when data did not meet the parametric assumptions. *p* < 0.05 indicated statistical significance, with *p* < 0.001 reflecting a more stringent threshold.

## 3. Results and Discussion

### 3.1. Effects of Y and C Exposure and DRE Intervention on the Intestine

#### 3.1.1. DRE Alleviated Intestinal Histological Injury in Mice Exposed to Y or C

**Figure 2:**
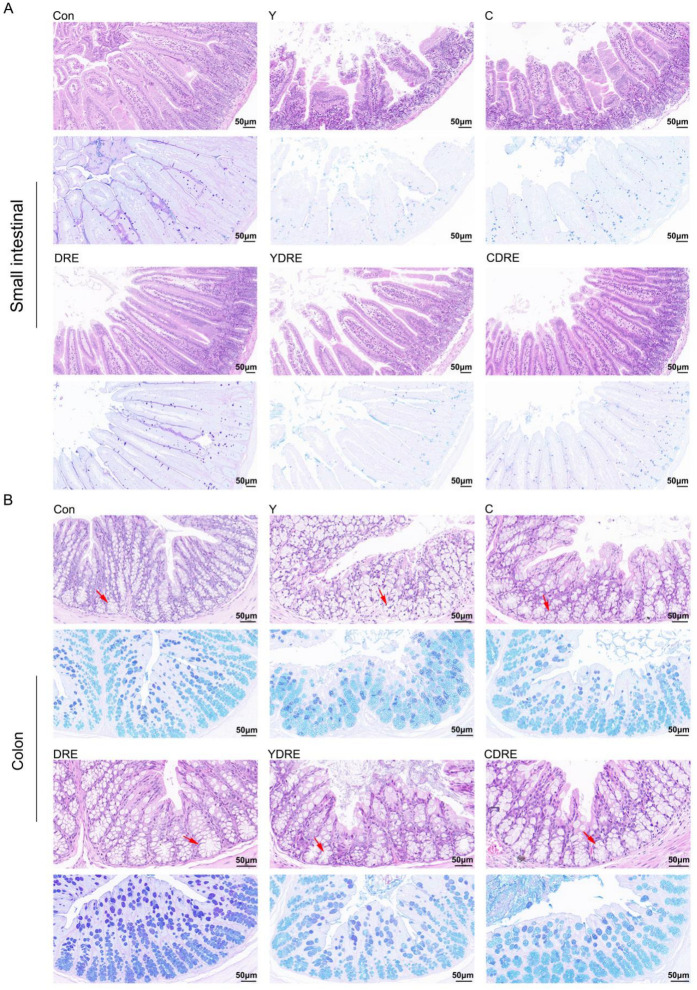
H&E and AB-PAS staining images showing the effects of DRE intervention on the pathology of the small intestinal and colon tissues of mice exposed to Y or C: (**A**) small intestine; (**B**) colon. Upper panel: H&E stained slides; lower panel: AB-PAS stained slides. Scale bar = 50 μm; the red arrows indicate the crypt structures.

Representative H&E staining results and histopathological scores of the mouse intestines (small intestine and colon) with or without intervention were illustrated in Figure 2 and Figure S7. Both Y and C damaged the villus structure of the small intestine, resulting in villus swelling, fewer villi with reduced lengths, and the destruction of the intestinal mucosal barrier. The damage caused by Y to the small intestine was significantly more severe than that caused by C (Figure 2, Figures S7 and S8), which was consistent with the findings of Zhang et al. [16]. Compared to the model group, DRE intervention significantly reduced the damage caused by Y and C to the small intestinal tissue, and its effects on Y-induced damage were particularly pronounced. As shown in Figure 2, after DRE intervention, the intestinal and villus swelling were significantly reduced, while the villus length and crypt depth were significantly increased. AB-PAS staining of the small intestine revealed substantially fewer goblet cells and less mucus secretion in the Y and C groups than in the control (Con) group, but DRE intervention did not significantly reverse the effects. Moreover, H&E staining revealed the impact of DRE intervention on the morphology and structure of the colon tissue in Y- and C-exposed mice (Figure 2 and Figure S8). The colon tissue structure of the Con group was normal and displayed neatly arranged villi and crypts and an intact epithelial structure. In contrast, both Y and C exposure resulted in a disordered arrangement of and structural damage to the colonic crypts and a decrease in the number of goblet cells. In addition, Y exposure led to epithelial shedding, cell dissolution, and necrosis of the colonic villi, whereas exposure to toxin C caused shorter villi (Figure 2). The DRE treatment restored the compact and well-ordered intestinal villous crypts, and AB-PAS staining revealed that mucus secretion was more abundant after DRE administration. Nevertheless, the effect of DRE on colonic mucus secretion in Y- and C-exposed mice was not significant (Figure 2). Overall, exposure to Y and C resulted in different manifestations of damage in the small intestine and colon, but DRE intervention significantly reduced the damage caused by these two toxins to these two intestinal segments. Furthermore, morphological changes in the villi and crypts can indicate the degree of intestinal damage [29]. Intestinal damage often leads to fewer epithelial cells. Crypt cells can replace damaged villus cells by differentiating into epithelial cells, but this further exacerbates intestinal damage, resulting in shorter villi and damaged crypts [30]. The above data, along with findings of Zhang et al. [16], effectively explained the mechanisms by which Y and C damage the intestine and DRE ameliorates such damage. Additionally, disruption of intestinal integrity might promote the localization of harmful factors to the liver via portal vein circulation [18], but DRE might reduce the transport and accumulation of toxins through the gut-liver axis by improving intestinal integrity and alleviating intestinal mucosal damage.

#### 3.1.2. DRE Alleviated Intestinal Inflammation in Mice Exposed to Y or C

**Figure 3:**
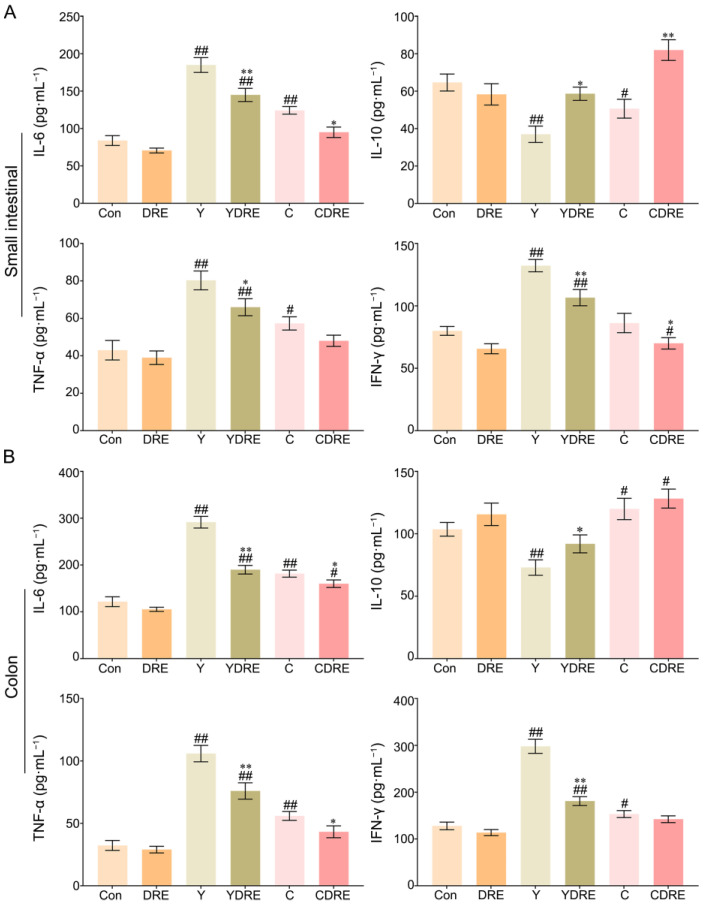
Effects of DRE intervention on the levels of the inflammatory factors IL-6, IL-10, TNF-α, and IFN-γ in the small intestinal and colon tissues of mice exposed to Y or C: (**A**) small intestine; (**B**) colon. The data are presented as the mean ± SD (n = 6). ^#^ Indicates a significant difference compared with the control group (Con) (^#^ *p* < 0.05; ^##^ *p* < 0.001). * Indicates a significant difference between the Y and YDRE groups or between the C and CDRE groups (* *p* < 0.05; ** *p* < 0.001).

IL-6, IL-10, TNF-α, and IFN-γ play critical roles in the immune system during inflammation [31]; therefore, the concentrations of each of these factors were measured in each group. As shown in Figure 3A and Table S1, compared to those in the Con group, the contents of IL-6 and TNF-α in the small intestines of the Y and C groups were significantly greater, whereas the content of IL-10 was significantly lower. In addition, the content of IFN-γ in the Y group was significantly greater than that in the Con group, while there was no significant difference in IFN-γ level between the C and Con groups. The increased expression of inflammatory factors and decreased expression of an anti-inflammatory factor in the Y group were more pronounced than those in the C group, which indicated that the inflammatory effects of Y on the small intestine were greater than those of C. DRE intervention significantly decreased the contents of IL-6 and IFN-γ in the small intestine of mice exposed to Y or C, while increasing the content of IL-10. Similarly, the content of TNF-α in the YDRE group was significantly lower than that in the Y group, but there was no significant difference in TNF-α level between the CDRE and C groups. Thus, the damage caused by Y and C to the colon cannot be ignored.

As shown in Figure 3B and Table S1, exposure to Y or C also significantly increased the levels of the inflammatory factors IL-6, TNF-α, and IFN-γ in colon tissue, with the consequences for the Y group being more dramatic than those in the C group. The degree of colon damage caused by these two byproducts was also mirrored by the colonic tissue morphology (Figure S8). Y and C downregulated and upregulated the anti-inflammatory factor IL-10, respectively, in colon tissue. After DRE intervention, IL-6, TNF-α, and IFN-γ expression significantly decreased and IL-10 expression significantly increased in the colons of the mice exposed to Y or C, but the differences in IL-10 or IFN-γ expression levels between the C and CDRE groups were insignificant. Overall, the above results indicated that these two harmful byproducts could promote intestinal damage in mice by causing a severe inflammatory response, and that Y was more toxic than C, which was in accordance with the pathological results (Figure 2). In addition, the downregulation of inflammatory factors and upregulation of anti-inflammatory factors confirmed the anti-inflammatory potential of DRE in vivo and its ability to alleviate the intestinal toxicity of the byproducts Y and C. Furthermore, the network toxicology prediction revealed that Y and C were highly correlated with the MAPK pathway genes, suggesting that the MAPK/p38 signaling pathway plays a critical role in the intestinal inflammation mediated by Y and C and that DRE might reduce the inflammation by inhibiting the MAPK/p38 signaling pathway [25].

#### 3.1.3. DRE Alleviated Intestinal Oxidative Stress in Mice Exposed to Y or C

**Figure 4:**
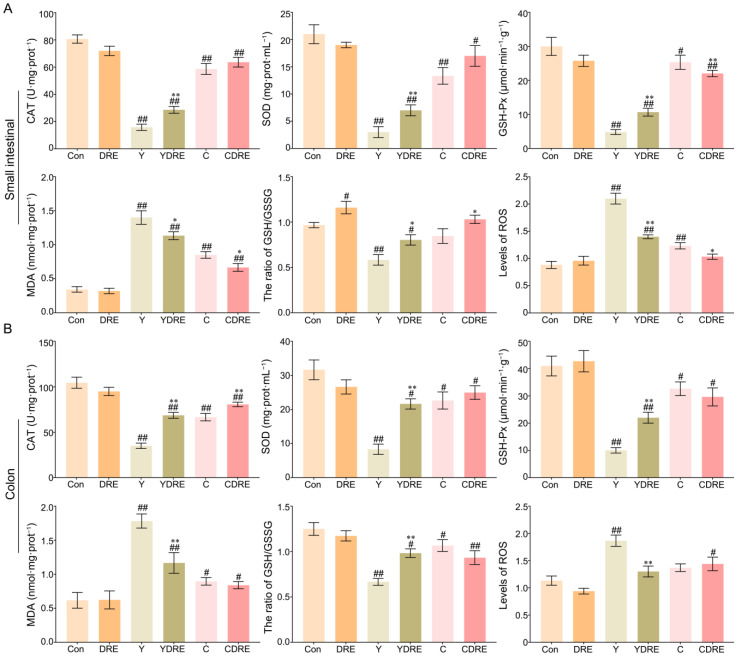
Effects of DRE intervention on the levels of oxidative stress biomarkers in the small intestinal and colon tissues of mice exposed to Y or C: (**A**) small intestine; (**B**) colon. The data are presented as the mean ± SD (n = 6). ^#^ Indicates a significant difference compared with the control group (Con) (^#^ *p* < 0.05; ^##^ *p* < 0.001). * Indicates a significant difference between the Y and YDRE groups or between the C and CDRE groups (* *p* < 0.05; ** *p* < 0.001).

Exogenous harmful factors lead not only to tissue damage but also to the production of excessive free radicals in tissues [28]. Oxidative stress is one of the pathogenic mechanisms of intestinal diseases and is thus an important indicator of tissue damage [32]. Therefore, the levels of CAT, SOD, GSH-Px, MDA, and ROS and the GSH/GSSG ratio, which are related to oxidative stress, were measured in intestinal tissues. As shown in Figure 4A and Table S2, significantly increased MDA levels and significantly decreased CAT, SOD, and GSH-Px contents were observed in the small intestines of the mice in the model group. However, DRE intervention significantly increased the CAT, SOD, and GSH-Px contents and significantly decreased the MDA level in the small intestine of Y-exposed mice. Notably, DRE intervention significantly reduced the contents of GSH-Px and MDA in the small intestine of C-exposed mice but had no major effects on CAT or SOD levels. Therefore, Y caused significantly more oxidative damage in the small intestine than did C, and the effects of DRE on oxidative stress in the small intestine caused by Y were significantly greater than those caused by C.

The colon is another important segment of the intestine, and its response to oxidative stress caused by these two byproducts cannot be overlooked. As shown in Figure 4B and Table S2, oxidative stress caused by Y and C in the colon was evaluated by detecting changes in the colon antioxidant defence system and the expression levels of oxidative stress markers. Colon analysis revealed that DRE did not significantly change the production of oxidative stress markers. Compared to those in the Con group, Y and C exposure led to significant decreases in the activities of CAT, SOD, and GSH-Px, as well as a significant increase in the content of MDA, confirming the occurrence of oxidative stress. In mice exposed to Y, DRE dramatically increased the levels of CAT, SOD, and GSH-Px in the colon and significantly reduced the content of MDA. With the exception of CAT, DRE intervention failed to significantly reduce the colonic oxidative stress in mice exposed to C. In addition, Y or C exposure also led to a decrease in the ratio of GSH–GSSG and an increase in the ROS level (Figure 4A,B), which also indicated that the antioxidant defence systems of the small intestine and colon were activated and that there was an imbalance in the production and scavenging of free radicals [28,33]. DRE intervention significantly decreased the level of ROS and increased the proportion of GSH in the small intestines of the Y and C groups and in the colon of the Y group but had no significant effect on the colon of the C group (Figure 4A,B). On the basis of the above results, it was concluded that immune and antioxidant defence systems were activated in response to Y or C administration, leading to intestinal injury, but DRE could protect against Y- and C-induced intestinal damage via anti-inflammatory and antioxidant mechanisms.

### 3.2. Effects of Y and C Exposure and DRE Intervention on the Liver

#### 3.2.1. DRE Alleviated Liver Histological Injury in Mice Exposed to Y or C

**Figure 5:**
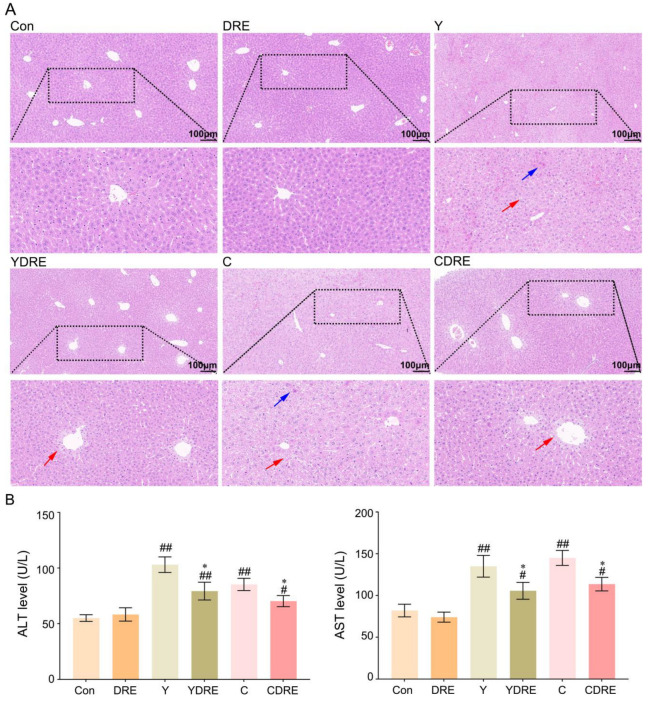
Effects of DRE intervention on liver histopathology and function in mice exposed to Y or C. (**A**) Representative images of H&E-stained liver tissues (scale bar: 100 µm). The blue and red arrows indicate necrosis and diffuse vacuolar degeneration of hepatocytes, respectively. (**B**) The activity of ALT and AST in serum. The data are presented as the mean ± SD (n = 6). ^#^ Indicates a significant difference compared with the control group (Con) (^#^ *p* < 0.05; ^##^ *p* < 0.001). * Indicates a significant difference between the Y and YDRE groups or between the C and CDRE groups (* *p* < 0.05).

As shown in Figure 5A and Figure S7, the liver structure of the mice in the Con and DRE groups was normal and displaying no obvious histological abnormalities. The livers of the mice exposed to Y or C exhibited obvious damage, characterized by disrupted liver structures, cells with different morphologies and sizes, obscured cell boundaries, and disordered arrangements of hepatic cords. Diffuse vacuolar degeneration of hepatocytes, the disappearance of multiple hepatocyte nuclei, and punctate necrosis of hepatocytes were also important phenotypes of the pathological changes in the liver. The consistency in characteristics of the phenotypes in the research of Wang et al. supported the generalizability and reproducibility of our results [34]. The livers of the mice in the YDRE and CDRE groups displayed certain histological damage as well, but the damage and lesions were less severe than those in the model group. Additional pathological changes included mild vacuolar degeneration, nuclear pyknosis, nuclear disappearance, and focal necrosis of hepatocytes, distributed mainly around the hepatic sinus space. DRE intervention reduced the liver damage caused by Y and C, indicating that *D. rubrovolvata* has good hepatoprotective effects, consistent with the reported protective effects of *D. rubrovolvata* aqueous extract against liver damage induced by carbon tetrachloride [23]. Lastly, destruction of the intestinal barrier facilitates the entry of harmful bacteria and endotoxins into the portal vein through intestinal epithelial cells, which might aggravate the hepatotoxicity of Y and C; however, DRE mitigated liver damage by inhibiting this process.

#### 3.2.2. DRE Intervention Decreased the Expression of Serum Liver Function Markers in Mice Exposed to Y or C

Serum levels of ALT and AST are critical biomarkers of liver injury [35]. As shown in Figure 5B and Table S3, compared with those in the control group, the levels of ALT and AST in the Y and C exposure groups were significantly greater, indicating liver injury. Elevated serum ALT and AST levels not only indicated significant hepatic injury but also suggested the occurrence of liver inflammation, as confirmed in the study by Wang et al. [34]. In addition, these two serum markers were significantly decreased in the YDRE and CDRE groups, demonstrating that DRE intervention effectively protected the liver from Y- and C-induced liver injury. The protective effect of DRE on the hepatotoxicity caused by drinking water disinfection byproducts was also reported in the study by Ye et al. [23].

#### 3.2.3. DRE Alleviated Liver Oxidative Stress in Mice Exposed to Y or C

**Figure 6:**
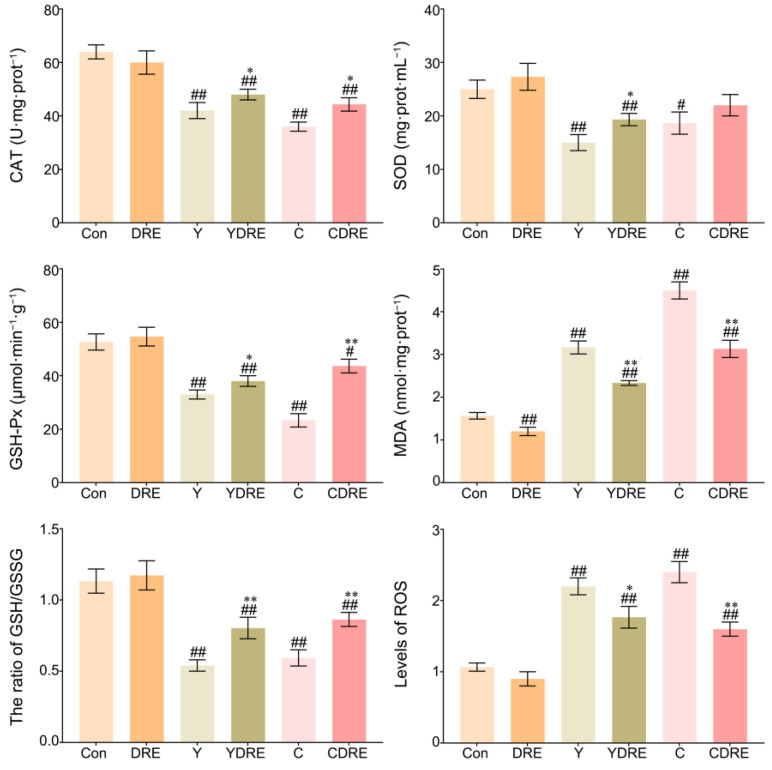
Effects of DRE intervention on the levels of oxidative stress biomarkers in the liver tissues of mice exposed to Y or C. The data are presented as the mean ± SD (n = 6). ^#^ Indicates a significant difference compared with the control group (Con) (^#^ *p* < 0.05, ^##^ *p* < 0.001). * Indicates a significant difference between the Y and YDRE groups or between the C and CDRE groups (* *p* < 0.05; ** *p* < 0.001).

ROS are key parameters reflecting the redox state of the body. When the production rate of ROS exceeds the scavenging rate of the antioxidant defence system in the body, ROS accumulates in a high level, which leads to severe oxidative stress in the body [36]. Figure 6 and Table S2 showed that the liver ROS levels in the Y and C exposure groups were significantly enhanced, while those in the livers of the DRE groups were significantly lower, indicating that these two byproducts caused an imbalance in the redox state of the liver and that DRE intervention significantly reduced ROS accumulation to restore it. As shown in Figure 6, the expression levels of CAT, SOD, and GSH-Px in the livers of the mice in the Y and C groups were significantly lower, whereas the activities of CAT, SOD, and GSH-Px in the livers of the YDRE and CDRE groups were significantly greater. These results indicated that these two byproducts impaired the antioxidant capacity of the liver, but DRE intervention effectively maintained the liver antioxidative defence system and improved the ability of the liver to scavenge ROS. In addition, the significant increases in MDA content caused by Y and C indicated that the liver tissue cells were severely affected by free radicals, but DRE intervention might effectively prevent the increase in MDA content by improving the redox status of liver tissue. Furthermore, the results also revealed that exposure to Y and C caused a significant decrease in the GSH content in the liver, whereas DRE intervention significantly increased the GSH level in the livers of the exposed mice. The protective effect of DRE on liver oxidative injury induced by Y and C might be closely related to the abundance of antioxidant substances, such as polysaccharides and polyphenols in the extract. Previous studies have confirmed that the extract of *D. rubrovolvata* has strong antioxidant capacity in vivo and in vitro and can effectively alleviate oxidative damage to the liver caused by harmful exogenous factors [21,24]. Furthermore, maintaining intestinal homeostasis and inhibiting the negative interactions between the intestine and liver might constitute another important pathway via which DRE mitigates Y- and C-induced liver oxidative damage.

### 3.3. DRE Alleviated Gut Dysbiosis in Mice Exposed to Y or C

**Figure 7:**
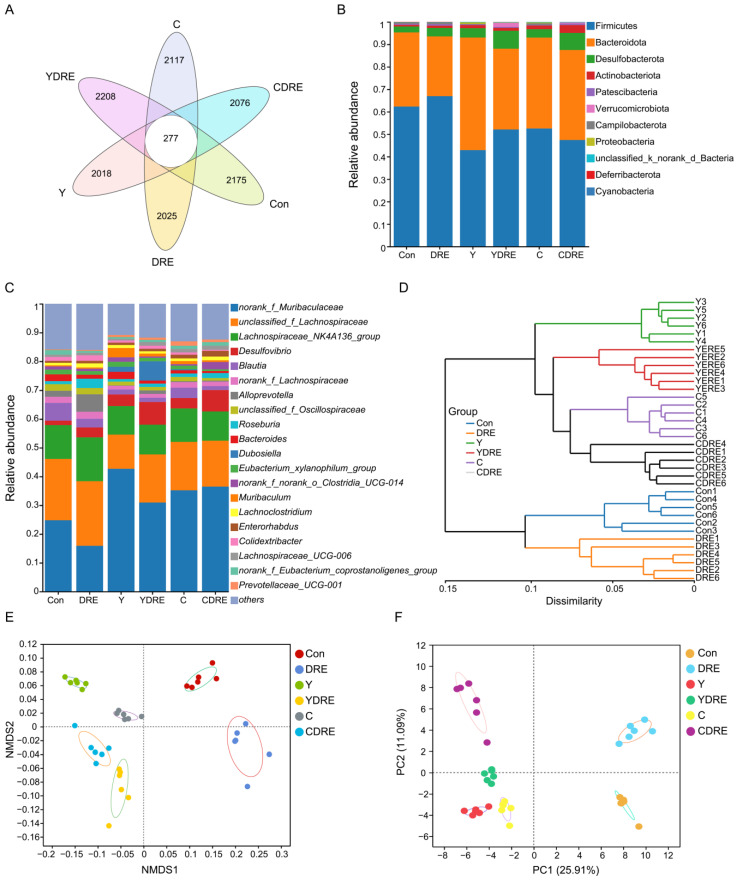
Effects of DRE intervention on the gut microbiome of mice exposed to Y or C (n = 6). (**A**) Venn diagram of the six groups at the ASV level. (**B**) Phylum-level bacterial community compositions of the six groups. (**C**,**D**) Gut microbiota composition and hierarchical clustering tree at the genus level between the six groups. (**E**,**F**) β diversity evaluated according to nonmetric multidimensional scaling (NMDS) analysis and principal component analysis (PCA) at the genus level.

The gut microbiota is a crucial sensitive indicator of the host response to harmful exogenous factors. Studies have shown that exposure to disinfection byproducts in drinking water leads to an imbalance in the intestinal microbiota, resulting in a reduction in beneficial bacteria and an increase in harmful ones [37]. To study the effects of the two byproducts on the changes in the intestinal microbiota and the effects of DRE intervention on these changes, 16S rRNA analysis was performed on the cecal contents of the mice. Cluster analysis revealed the common and unique ASVs from various groups using a Venn diagram (Figure 7A). A total of 12,896 ASVs were obtained, of which 277 ASVs were shared by six groups, and 2175, 2025, 2018, 2208, 2117, and 2076 ASVs were unique to the Con, DRE, Y, YDRE, C, and CDRE groups, respectively. There were no significant differences in the α diversity indices (Ace, Chao, Sobs, and Simpson) among the groups at the ASV level (Figure S9), indicating that exposure to the two byproducts and DRE intervention did not lead to significant changes in intestinal microbial richness or diversity. The ASVs were then analyzed to determine the relative abundances of bacteria at different taxonomic levels, such as the phylum and genus levels. Firmicutes was the predominant phylum, accounting for 62.30%, 66.93%, 42.89%, 52.07%, 52.53%, and 47.40% of the gut microbiota in the Con, DRE, Y, YDRE, C, and CDRE groups, respectively, followed by Bacteroidetes, accounting for 33.02%, 26.62%, 50.17%, 36.00%, 40.50%, and 40.10%, respectively (Figure 7B). An imbalance in the ratio of Firmicutes to Bacteroidetes in the intestine is closely related to the occurrence of various intestinal diseases. For example, studies have reported that the Firmicutes–Bacteroidetes ratio in the intestines of patients with ulcerative colitis is significantly decreased [38]. In this study, Y and C exposure, particularly Y exposure, resulted in a significant decrease in the ratio of Firmicutes to Bacteroidetes in the intestines of the mice, and the degree of destruction of intestinal flora homeostasis caused by Y and C was consistent with the inflammation and oxidative stress observed in the intestine (Figure 3, Figure 4, and Figure 7B). An imbalance in the intestinal flora, in turn, aggravates the damage to intestinal barrier function, immune function, and antioxidant function and then damages the liver through the liver–gut axis [18]. DRE intervention significantly increased the ratio of Firmicutes to Bacteroidetes in the intestines of Y-exposed mice but barely changed the ratio in C-exposed mice, which might be associated with intestinal inflammation and oxidative stress. In addition, *norank_f_Muribaculaceae*, *unclassified_f_Lachnospiraceae*, and *Lachnospiraceae_NK4A136_group* were the predominant genera in the six groups (Figure 7C). Moreover, Y and C exposure decreased the abundances of several probiotics, such as *unclassified_f_Lachnospiraceae* and *Lachnospiraceae_NK4A136_group*, while DRE intervention reversed these changes to a certain extent. We speculated that DRE might alleviate Y- and C-induced intestinal damage by altering the abundance of these beneficial bacteria, which were related to short-chain fatty acid (SCFA) production and anti-inflammatory effects [18]. Wang et al. demonstrated that disruption of gut microbiota composition and balance induced by Y and C exposure was closely associated with alterations in intestinal short-chain fatty acid levels, which aligned with the aforementioned findings [34]. Lastly, the specific relative abundances of the dominant bacterial genera in the gut microbiota were thoroughly analyzed (Figure S10). Taken together, the above results indicated that both Y and C exposure could lead to changes in the composition and structure of the gut microbiota. DRE intervention also changed the structure of the intestinal microbiota, promoting the remodeling of the intestinal microecology in mice exposed to Y or C.

**Figure 8:**
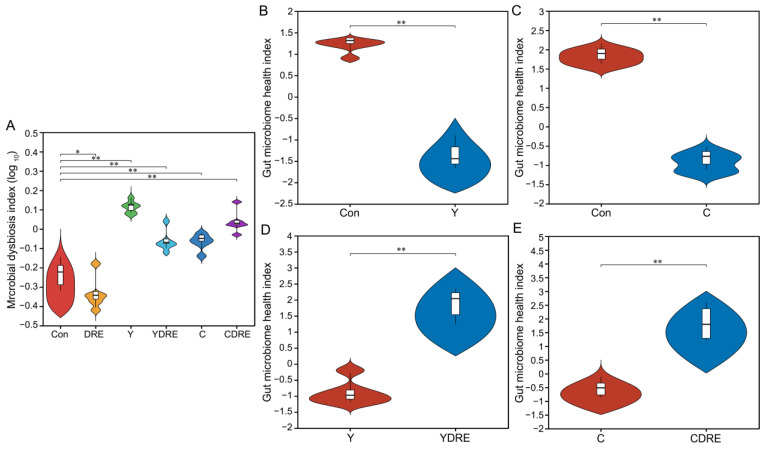
Effects of DRE intervention on the alteration of the gut microbiota phylotype and the severity of gut microbiota dysbiosis in mice exposed to Y or C (n = 6). (**A**) Violin plots displaying the median and quartiles of the microbial dysbiosis index (MDI). (**B**–**E**) Violin plots displaying the median and quartiles of the gut microbiome health index (GMHI). Significance was determined by the Wilcoxon rank sum test method. * Represents a difference between groups (* *p* < 0.05; ** *p* < 0.001).

A total of 36 samples from the six groups were divided into four categories via hierarchical cluster analysis at the genus level, among which Con and DRE constituted one category, C and CDRE another, and Y and YDRE a third (Figure 7D). ANOSIM, NMDS, PCA, and PCoA were performed to analyze the β diversity, and, as shown in Figure 7E,F and Figure S11, the microbial landscapes of the six groups were significantly different, and there was distinct separation of the cluster of each group. Furthermore, to evaluate the gut microbiota imbalance in each group, the microbial dysbiosis index (MDI) was obtained by combining the relevant taxa of each group at the genus level. Compared with that of the Con group, the MDI value of the DRE group was significantly lower; that was, while the degree of gut dysbiosis was lower, the intestinal homeostasis was greater. The gut microbiota of the mice exposed to Y and C, particularly Y, presented relatively high MDI values, and DRE intervention significantly improved the increase in the MDI caused by Y but failed to alleviate the imbalance in the microbiota caused by C (*p* < 0.001) (Figure 8A). To further assess disease correlation among the treatments, the gut microbiome health index (GMHI) was used to evaluate health status (Figure 8). Similar to the MDI results, Y- and C-exposed mice were more likely to have lower GMHI values (Figure 8B,C), but DRE intervention caused Y- and C-exposed mice to be more likely to have higher GMHI values (Figure 8D,E). That is, Y and C exposure might cause high disease risk, and DRE intervention might reduce this risk. Collectively, the above results suggest that exposure to the two byproducts Y and C resulted in significant changes in the composition and structure of the gut microbiota. DRE intervention also changed the structure of the intestinal microbiota and alleviated the gut dysbiosis caused by Y and C. DRE more effectively improved Y-induced intestinal injury, which aligns with the histological and physicochemical test results. These data confirm that an imbalance in the composition and diversity of the gut microbiota is closely related to intestinal histological damage, an activated inflammatory response and oxidative stress [39].

### 3.4. Evaluation of Integrated Biomarker Response and Correlation Analysis of Intestine and Liver

#### 3.4.1. Integration of Biomarker Responses

**Figure 9:**
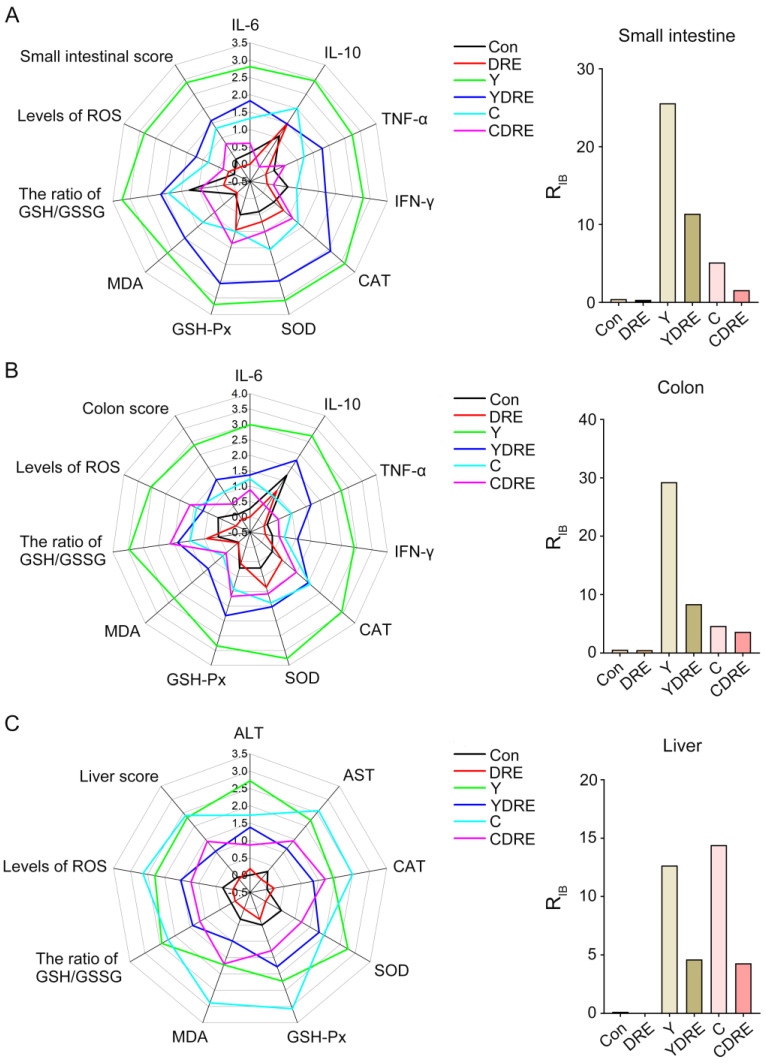
The integrated biomarker response analysis of small intestine, colon and liver biomarkers. The radar diagram of IBR and R_IB_ values of IBR on small intestine (**A**), colon (**B**), and liver tissue (**C**).

In order to intuitively analyze the overall response of the intestine and liver under the action of the disinfection byproducts and/or DRE, and to further verify the reliability of the biochemical analysis results, the data of the biochemical marker were estimated with the integrated biomarker response index (IBR). The impact of pollutants on organisms was expressed by the R_IB_ value, and the greater the value, the more serious the impact [28]. The corresponding IBR levels and R_IB_ values of small intestine, colon, and liver tissues are shown in Figure 9 and Table S4. Regardless of the small intestine and colon, the R_IB_ values of the Y group were higher than those of the C group. The R_IB_ value of the disinfection byproducts exposure groups under the premise of DRE intervention was significantly lower than that of the exposure groups without intervention. For the liver, the R_IB_ value of C group was slightly larger than that of Y group, and DRE intervention could significantly down-regulate the R_IB_ value of the exposure groups. The above results demonstrated that Y induced more severe intestinal damage than C, while the opposite was observed in liver tissue. DRE intervention effectively mitigated the intestinal and liver toxicity of the two disinfection byproducts through multiple pathways, including alleviating histopathological lesions, suppressing inflammatory responses, and enhancing tissue antioxidant capacity. These findings were consistent with and corroborated the physicochemical analysis results. Anti-pathological damage and antioxidant and anti-inflammatory effects constituted the key molecular pathways through which the extract alleviated disinfection byproduct-induced tissue damage [40]. These three mechanisms operated synergistically through complex signaling networks, collectively forming the core of the cytoprotective mechanism. Current research demonstrated that active components in the extract, such as polysaccharides and polyphenols, could mitigate oxidative stress damage through both direct free radical scavenging and upregulation of the Nrf2/ARE pathway [41]. Concurrently, these components could inhibit the NF-κB and MAPK signaling pathways, downregulate the release of pro-inflammatory cytokines (e.g., TNF-α, IL-6), and consequently, block inflammatory cascades [42]. Notably, pathological damage, oxidative stress, and inflammatory responses exhibited positive feedback regulation (e.g., ROS-mediated activation of the NLRP3 inflammasome), and natural extracts could achieve synergistic effects via multi-target modulation [40,43]. This multi-target interactive mechanism provided a theoretical foundation for developing natural extract-based therapeutic strategies against DBP-induced toxicological damage.

#### 3.4.2. Correlation Between Intestinal and Liver Injury Markers and Intestinal Microflora

**Figure 10:**
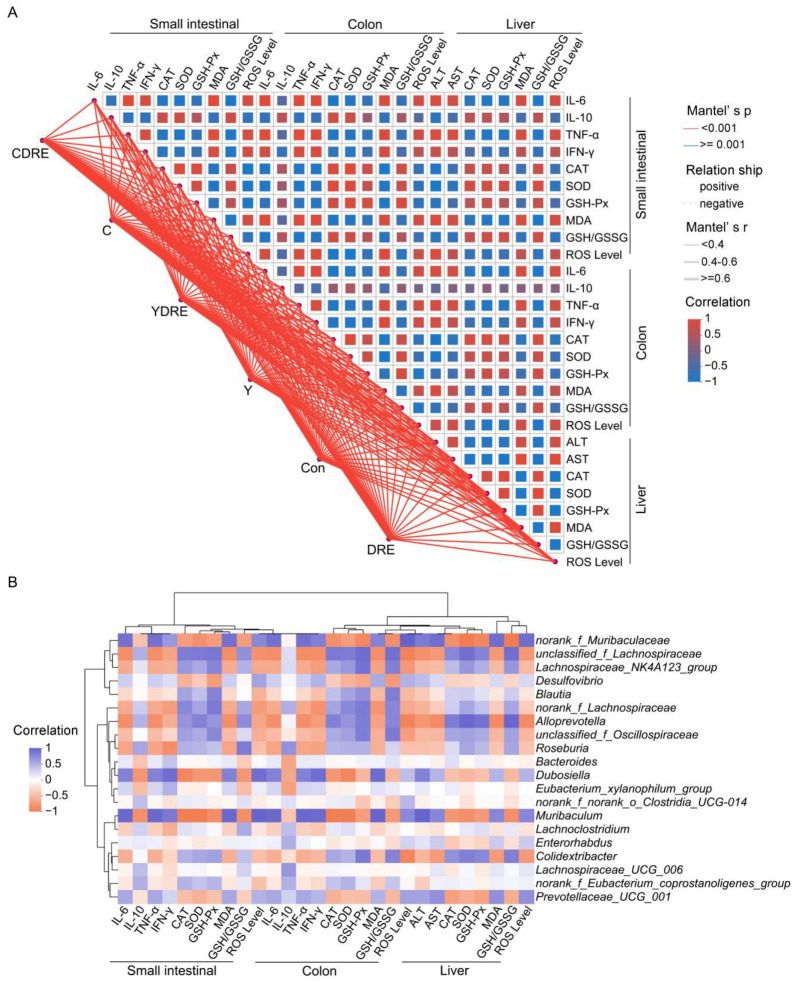
Spearman’s correlation analysis. (**A**) Correlation network heatmap of the 28 biochemical indicators. Mantel’s r indicates the threshold of the absolute value of the correlation between two distance matrices in the Mantel test; Mantel’s *p* indicates the threshold of the *p* value in the Mantel test; and Correlation represents the Spearman correlation coefficient. (**B**) Correlation heatmap of the top 20 genera and 28 biochemical indicators.

On the basis of the Spearman correlation coefficient and Mantel test results, a correlation network heatmap was generated for data-driven correlation analysis between each treatment, along with 28 biochemical indicators reflecting intestinal and liver physiology, oxidative stress, and inflammation, to further explore the synergistic effects of each treatment on intestinal and liver injury in mice. As shown in Figure 10A, the Mantel test confirmed that each treatment was significantly positively associated with intestinal and liver physiology, oxidative stress, and inflammation-related indicators. Spearman’s correlation analysis explored the interrelationships among the small intestine, colon, and liver physiology, oxidative stress, and inflammation-related indicators, highlighting significant correlations among the 28 biochemical indicators (Figure 10A). Specifically, these 28 biochemical indicators were strongly correlated with Y and C exposure and DRE intervention, with significant connections observed between indicators of the small intestine, colon, and liver. This study demonstrated the strong relationship between disinfection byproduct exposure and intestinal and liver physiology, oxidative stress, and inflammation [37]. In addition, as the first site exposure to harmful exogenous factors after oral ingestion, the intestine serves as a vital interface between the body and the external environment [44]. The intestine and liver participate in close and frequent crosstalk mediated via immunity and oxidative stress. For example, the portal vein blood discharged from the mesenteric vein flows into the liver, so the liver is a crucial organ exposed to invading factors from the intestine [45]. From this perspective, both the intestinal and liver damage caused by Y and C exposure, as well as the alleviation of damage caused by DRE intervention, are inseparable from the intestine–liver axis. Moreover, polysaccharides and polyphenols of edible fungi are key active substances in alleviating the liver and intestinal damage caused by harmful exogenous factors [46]. In our previous research, the *D. rubrovolvata* extraction process was optimized to significantly increase the contents of polysaccharides and polyphenols in the extract, thus significantly enlarging its various antioxidant capacities [21]. As shown in Figure 10A, the antioxidant effects of DRE on the liver and intestine were significantly positively correlated with its anti-inflammatory effect. Therefore, the protective effects of DRE against liver and intestinal injury caused by Y and C might largely be attributed to the abundant antioxidant polysaccharides and polyphenols in the extract.

Spearman’s correlation analysis revealed that *norank_f_Muribaculaceae*, *Dubosiella*, *Muribaculum*, and *Prevotellaceae_UCG_001* were negatively correlated with intestinal and liver inflammation and oxidative stress indices (IL-10, CAT, SOD, and GSH-Px levels, and the GSH/GSSG ratio). *Unclassified_f_Lachnospiraceae*, *Lachnospiraceae_NK4A123_group*, *Blautia*, *norank_f_Lachnospiraceae*, *Alloprevotella*, *unclassified_f_Oscillospiraceae*, *Roseburia*, and *Colidextribacter* were negatively correlated with inflammatory factors, oxidative damage, and liver function damage indicators (IL-6, TNF-α, IFN-γ, MDA, ROS, ALT, and AST) (Figure 10B). As metabolites of the intestinal flora, SCFAs, such as butyric acid, are regulated by the intestinal microbiota, participate in the regulation of host immunity and inflammation, and play important roles in enterohepatic circulation [18]. As a butyric acid-producing bacterium, *Roseburia* can improve intestinal biodiversity and plays important roles in controlling inflammation, especially intestinal inflammation. Furthermore, dietary supplementation with plant polysaccharides and polyphenols can boost the production of butyric acid and reduce the occurrence of inflammation [47,48,49]. Previous research results confirmed that DRE was rich in dietary polysaccharides and polyphenols [21], which might be important for the increased abundance of *Roseburia* and inhibition of Y- and C-mediated intestinal inflammation observed upon DRE intervention (Figure 7C and Figure 10B). These results again suggest that DRE could alleviate the intestinal and liver damage induced by Y and C and that modulation of the intestinal microbiota might be one of its pivotal mechanisms.

## 4. Conclusions

In this study, histopathological, molecular biology, and intestinal microbiome analyses were employed to systematically study the toxicity of the MET chlorination byproducts Y and C to the small intestine, colon, and liver and to explore the ability of DRE intervention to ameliorate this toxicity. Both Y and C exposure could disrupt intestinal homeostasis by destroying the intestinal structure, activating intestinal inflammation, inducing oxidative stress, and altering the intestinal microflora. Notably, the toxic effects of Y on the small intestine and colon were significantly stronger than those of C. Moreover, both Y and C exposure caused liver cell damage, structural abnormalities, and dysfunction and oxidative stress. In addition, DRE intervention effectively ameliorated the intestinal and liver damage induced by Y and C and optimized the intestinal microflora. Overall, this work provides a comprehensive understanding of the tissue toxicity of Y and C from multiple perspectives. It enhances knowledge of the toxic effects of disinfection byproducts in mammalian models and sheds light on the role of the microbiota–gut–liver axis in mediating these effects, thus providing new insights into practicable and efficacious prevention strategies.

## Data Availability

All data generated or analyzed during this study are included in this article.

## Supplementary Materials

The following supporting information can be downloaded at: https://www.mdpi.com/article/10.3390/toxics13040310/s1, Figure S1: UV–Vis spectra of metformin and the byproducts Y and C; Figure S2: FTIR spectra of metformin, Y and C; Figure S3: H-NMR spectra of byproducts Y and C; Figure S4: Severe pathological changes were observed in the small intestines and colons of the mice given a single injection of the byproducts Y or C; Figure S5: Severe pathological changes were observed in the livers of mice given a single injection of the byproducts Y or C; Figure S6: Effects of different DRE intervention doses on the levels of the oxidative stress biomarker MDA in the small intestine and the activity of ALT in serum; Figure S7: Histogram of the tissue injury scores; Figure S8: Changes in the morphology of the intestine; Figure S9: α diversity of the gut microbiota displayed by the Ace, Chao, Sobs and Simpson indices at the ASV level; Figure S10: Relative abundances of microorganisms at the genus level; Figure S11: Principal coordinate analysis (PCoA) based on the ANOSIM method at the genus level; Table S1: Levels of cytokines in the small intestines and colons of the mice; Table S2: Levels of oxidative stress indicators in the small intestines, colons and livers of the mice; Table S3: Levels of serum biochemical indicators reflecting liver function in the mice; Table S4: The scores of the integrated biomarker response (IBR).
