# Supplementary material for: Protective Effect of Dictyophora rubrovolvata Extract on Intestinal and Liver Tissue Toxicity Induced by Metformin Disinfection Byproducts
Source: Toxics. 2025 Apr 16;13(4):310. doi: 10.3390/toxics13040310 (PMC12030868; doi:10.3390/toxics13040310)
Supplement: Supplementary file 1 [file toxics-13-00310-s001.zip › Supplementary material.pdf]

Supplementary Information

Characterization of the prepared Y and C

The absorption spectra of MET and its two byproducts, Y and C, in the wavelength range of 200–600 nm acquired with a UV–Vis spectrophotometer. The data interval was 2 nm, and the size of the quartz cuvette was 1 mL. Strong characteristic absorption peaks of MET, Y and C appeared at 233, 390 and 240 nm, respectively (Fig. S1). Infrared spectra of MET, Y and C in the range of 400-4000 cm^{-1} under nitrogen protection. The C≡N stretching vibrations at 2201 cm^{-1} and 2151 cm^{-1} were characteristic peaks of compound C, which were significantly different from the characteristic peaks of Y (Fig. S2). The H-NMR spectra of byproducts Y and C in the range of 0–20 ppm acquired with a Bruker (600 MHz) nuclear magnetic resonance spectrometer (liquid), in which deuterated water (D_2O) and 3-(trimethylsilyl) propionic-2,2,3,3-d4 acid sodium salt (TSP-d4) were selected as the solvent and internal standard, respectively. In the spectra, the TSP-d4 singlet was located at -0.10 ppm; the two strong singlets of Y were located at 3.72 ppm and 3.33 ppm; and the two strong singlets of C were located at 3.24 and 3.11 ppm (Fig. S3). These singlets indicated that there were no interchangeable adjacent hydrogen atoms in byproducts Y and C. The purities of Y and C were determined on the basis of the integrated peak areas of the hydrogen atoms and the relevant information from the internal standard TSP-d4 (He et al., 2022).

$$P(\%) = \frac{\dfrac{m_I}{M_I \times V_I}}{\dfrac{m_x}{M_x \times V_x}} \times \frac{A_x}{A_I} \times 3 \times 100 \qquad (1)$$

In this equation, P refers to the purity (%) of compound x; m_I, M_I, V_I, and A_I refer to the mass, molar mass, solution volume and peak area of the internal standard; and m_x, M_x, V_x and A_x refer to the mass, molar mass, solution volume and peak area of compound x, respectively.

Fig. S1: UV–Vis spectra of metformin (black) and the byproducts Y (red) and C (blue); the wavelength range was 200–600 nm.

Fig. S2: FTIR spectra of metformin (black), Y (red) and C (blue). The absorption band near 2200 cm^{-1} corresponds to the C≡N stretching vibration.

Fig. S3: H-NMR spectra of byproducts Y and C. (A) Y. (B) C. The solvent was heavy water, and 3-(trimethylsilyl) propionic-2,2,3,3-d4 acid sodium salt (TSP-d4) was used as the internal standard. The characteristic peaks of TSP-d4, Y and C appeared at -0.10 ppm, 3.33 and 3.72 ppm, and 3.11 and 3.24 ppm, respectively.

Determination of the Y and C exposure doses

First, the mice were given a single injection of Y (50 mg/kg) or C (100 mg/kg), and the dose was selected according to the methods of Zhang et al. (2021). The outcomes of the mice were consistent with those of Zhang et al. (2021); that was, all of the mice in Y-exposed group died, whereas all the C-exposed mice survived. H&E staining of the small intestine, colon and liver revealed the key characteristics of intestinal and liver damage caused by short-term high-dose exposure to Y and C. This information could serve as an important basis for evaluating whether DRE could effectively alleviate the intestinal and liver damage caused by Y or C. Structural damage to the intestinal villus and crypt, cell shedding and necrosis, liver vacuolar lesions and hepatocyte necrosis were typical manifestations of short-term high-dose exposure to these two byproducts (Fig. S4 and S5). After careful consideration, the exposure dosages of Y and C were reduced to 2 mg/kg/d and 10 mg/kg/d, respectively, and the compounds were injected continuously for three days. This prevented interference caused by an excessive single injection dose and also ensured that the mice in the exposure groups had a normal diet and activity and

obvious pathological characteristics, which were used to intuitively observe effects of DRE.

Fig. S4: Severe pathological changes were observed in the small intestines (A) and colons (B) of the mice given a single injection of the byproducts Y or C (scale bar = 100 μm). The control group was injected with PBS, and Y and C were administered at doses of 50 mg/kg and 100 mg/kg, respectively.

Fig. S5: Severe pathological changes were observed in the livers of mice given a single injection of the byproducts Y or C (scale bar = 100 μm). The control group was injected with PBS, and Y and C were administered at doses of 50 mg/kg and 100 mg/kg, respectively. The blue and red arrows indicate necrosis and vacuolar degeneration of hepatocytes, respectively.

Determination of the DRE intervention dose

An important manifestation of intestinal damage caused by disinfection byproducts in drinking water is the induction of oxidative stress. Moreover, the excellent antioxidative performance of DRE has been widely confirmed and verified and improved in our previous research. Therefore, when screening the optimal dose for DRE intervention, MDA, an important marker of oxidative stress, was selected as one of the evaluation criteria to explore whether the two disinfection byproducts Y and C could cause oxidative stress in the body and whether DRE could prevent this oxidative damage. In addition, the extract of *D. rubrovolvata* effectively alleviated liver function damage caused by carbon tetrachloride, and Zhang et al. (2023) confirmed that Y and C exposure could cause abnormal liver function in mice. Therefore, ALT was used as another criterion to assess whether DRE intervention could alleviate liver damage caused by Y and C and to determine an effective intervention dose that could significantly improve liver damage. The preliminary exploration results indicated that the medium dose of the extract could protect intestine and liver

(Fig. S6), so a dosage of 56 mg/kg/d was selected for further experiments.

Fig. S6: Effects of different DRE intervention doses on the levels of the oxidative stress biomarker MDA in the small intestine (A) and the activity of ALT in serum (B). Con, Y, YDRED, YDREM, YDREG, C, CDRED, CDREM and CDREG refer to the control group, Y exposure group, Y exposure + DRE low-dose intervention group, Y exposure + DRE medium-dose intervention group, Y exposure + DRE high-dose intervention group, C exposure group, C exposure + DRE low-dose intervention group, C exposure + DRE medium-dose intervention group, and C exposure + DRE high-dose intervention group, respectively. Y and C exposure: 2 mg/kg/d Y, 10 mg/kg/d C, continuous injection for three days. Low, medium and high DRE intervention doses: 28, 56 and 84 mg/kg/d, respectively. The data are presented as the mean ± SD (n = 6). # indicates a significant difference compared with the Con group ($^{\#}P < 0.05$, $^{\#\#}P < 0.001$). * indicates a significant difference between the Y group and its three intervention groups or between the C group and its three intervention groups ($^{*}P < 0.05$, $^{**}P < 0.001$).

Fig. S7: Histogram of the tissue injury scores. (A) Small intestine scores. (B) Colon scores. (C) Liver scores. The data are presented as the mean ± SD (n = 6). # indicates a significant difference compared with the control group (Con) ($^{\#}P < 0.05$, $^{\#\#}P < 0.001$). * indicates a significant difference between the Y and YDRE groups or between the C and CDRE groups ($^{*}P < 0.05$, $^{**}P < 0.001$).

Fig. S8: Changes in the morphology of the intestine. The red arrows and blue arrows point to the typical sites of injury in the small intestinal and colon tissues, respectively.

Fig. S9: α diversity of the gut microbiota displayed by the Ace, Chao, Sobs and Simpson indices at the ASV level.

Fig. S10: Relative abundances of microorganisms at the genus level. The data are presented as the mean ± SD (n = 6). # indicates a significant difference compared with the control group (Con) ($^{\#}P$ < 0.05, $^{\#\#}P$ < 0.001). * indicates a significant difference between the Y and YDRE groups or between the C and CDRE groups (*P < 0.05, **P < 0.001).

Fig. S11: Principal coordinate analysis (PCoA) based on the ANOSIM method (999 permutations) at the genus level.

Table S1: Levels of cytokines in the small intestines and colons of the mice.

Table S2: Levels of oxidative stress indicators in the small intestines, colons and livers of the mice.

Table S3: Levels of serum biochemical indicators reflecting liver function in the mice.

Table S4: The scores of the integrated biomarker response (IBR).

Fig. S1

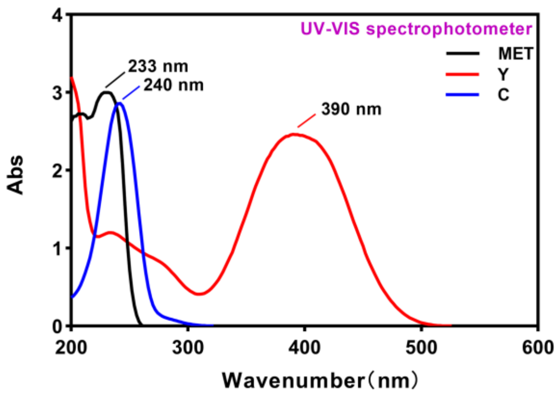
UV-VIS spectrophotometer
MET
Y
C
233 nm
240 nm
390 nm
Abs
Wavenumber（nm）
4
3
2
1
0
200
300
400
500
600

Fig. S2

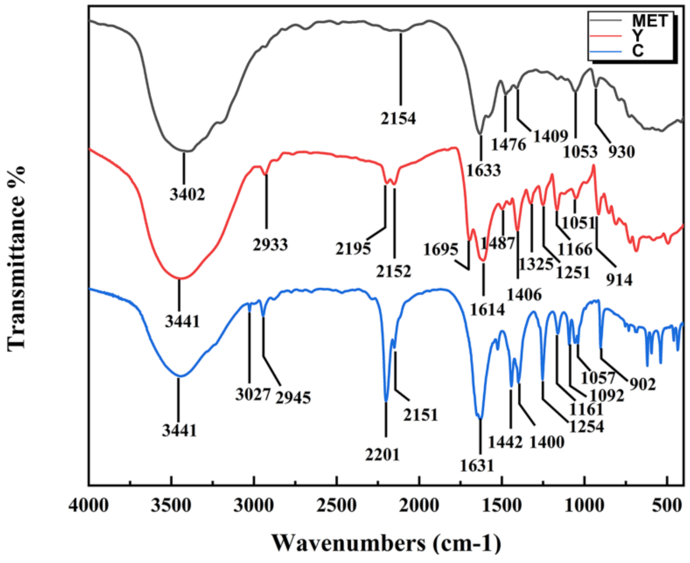
MET
Y
C
Transmittance %
Wavenumbers (cm-1)
4000
3500
3000
2500
2000
1500
1000
500
2154
3402
1633
1476
1409
1053
930
2933
3441
2195
2152
1695
1614
1487
1406
1325
1251
1166
1051
914
3027
2945
3441
2201
2151
1631
1442
1400
1254
1161
1092
1057
902

Fig. S3

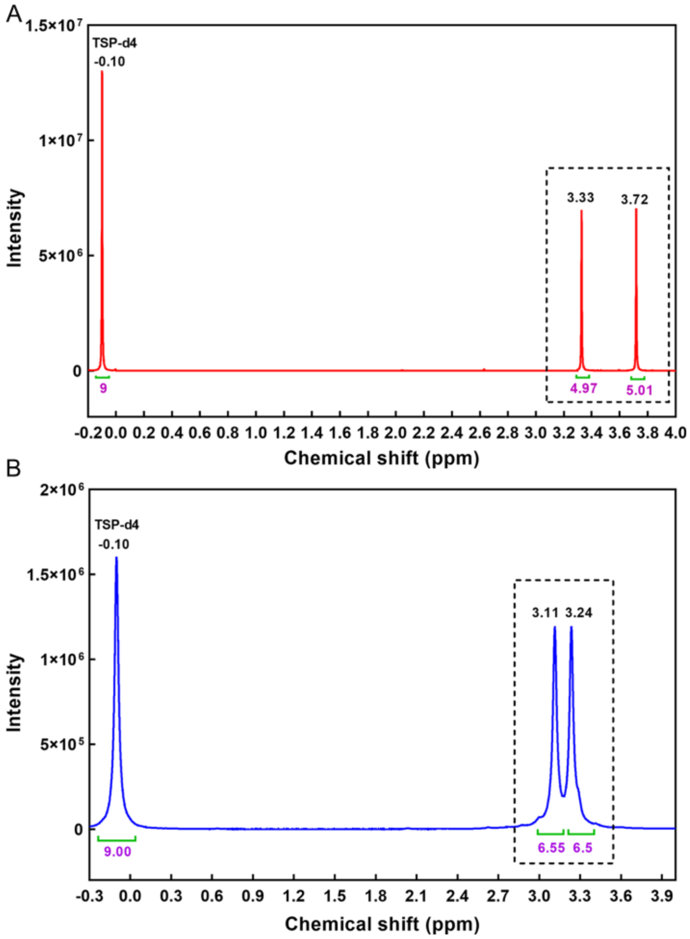
A
1.5×10^7
1×10^7
5×10^6
0
TSP-d4
-0.10
3.33
3.72
9
4.97
5.01
Intensity
-0.2 0.0 0.2 0.4 0.6 0.8 1.0 1.2 1.4 1.6 1.8 2.0 2.2 2.4 2.6 2.8 3.0 3.2 3.4 3.6 3.8 4.0
Chemical shift (ppm)
B
2×10^6
1.5×10^6
1×10^6
5×10^5
0
TSP-d4
-0.10
3.11
3.24
9.00
6.55
6.5
Intensity
-0.3 0.0 0.3 0.6 0.9 1.2 1.5 1.8 2.1 2.4 2.7 3.0 3.3 3.6 3.9
Chemical shift (ppm)

Fig. S4

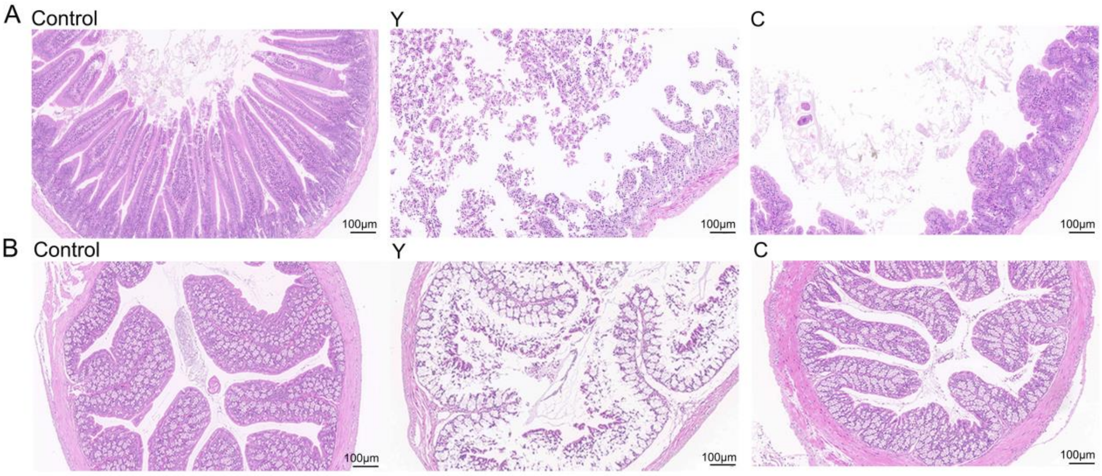
A
Control
100μm
Y
100μm
C
100μm
B
Control
100μm
Y
100μm
C
100μm

Fig. S5

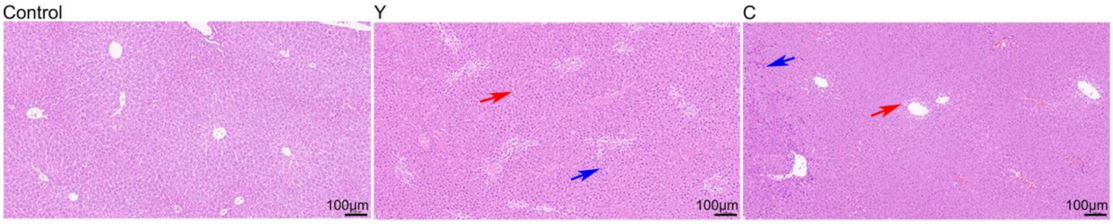

Fig. S6

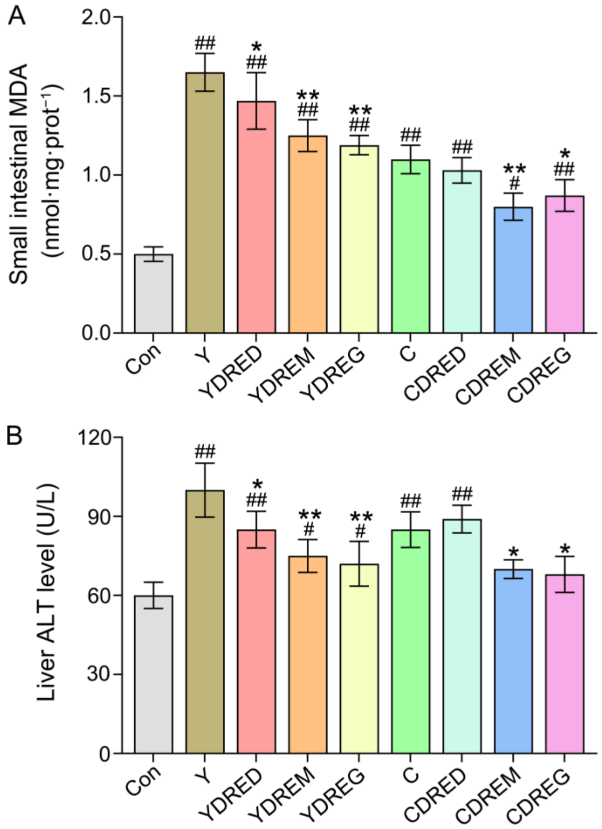

A
Small intestinal MDA (nmol·mg·prot⁻¹)
2.0
1.5
1.0
0.5
0.0
Con
Y
YDRED
YDREM
YDREG
C
CDRED
CDREM
CDREG
B
Liver ALT level (U/L)
120
90
60
30
0
Con
Y
YDRED
YDREM
YDREG
C
CDRED
CDREM
CDREG

Fig. S7

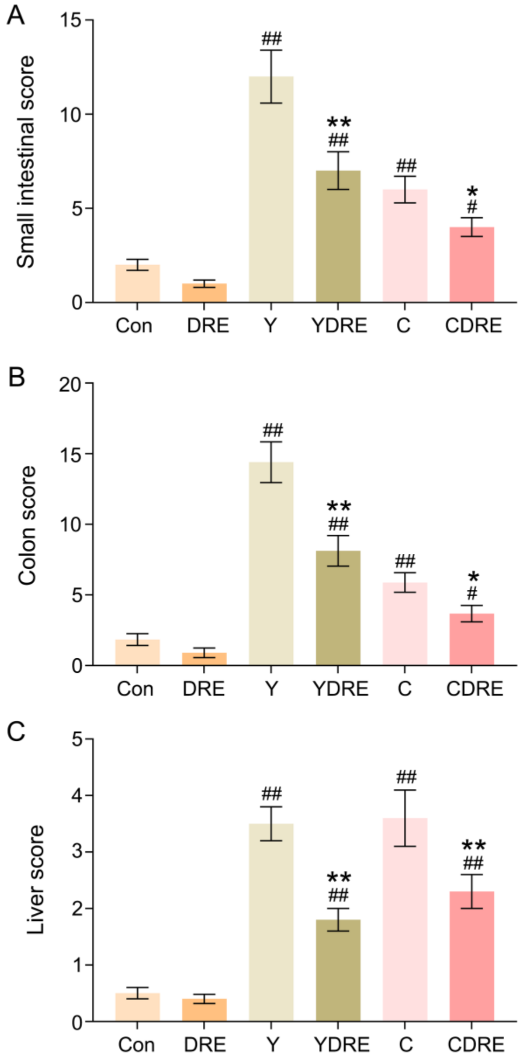
A
Small intestinal score
15
10
5
0
##
**
##
##
*
#
Con
DRE
Y
YDRE
C
CDRE
B
Colon score
20
15
10
5
0
##
**
##
##
*
#
Con
DRE
Y
YDRE
C
CDRE
C
Liver score
5
4
3
2
1
0
##
##
**
##
**
##
Con
DRE
Y
YDRE
C
CDRE

Fig. S8

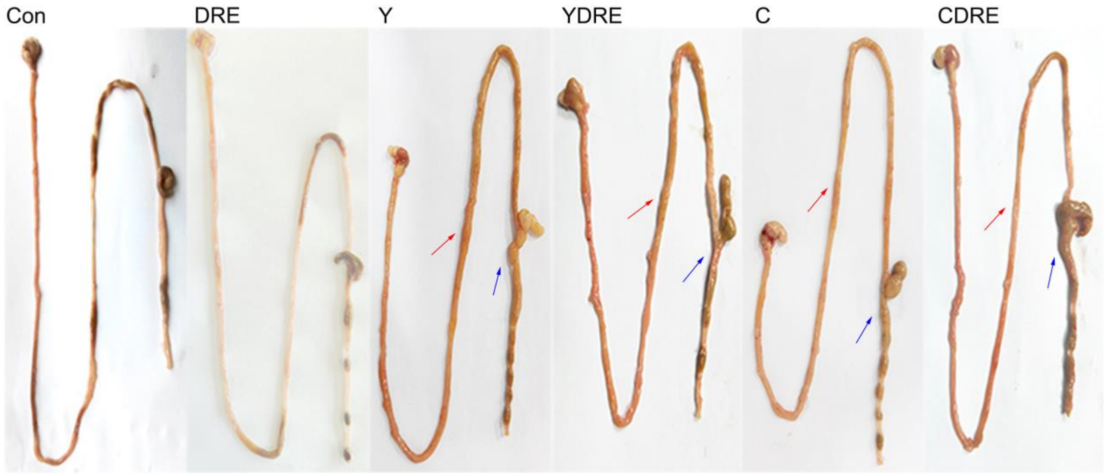

Fig. S9

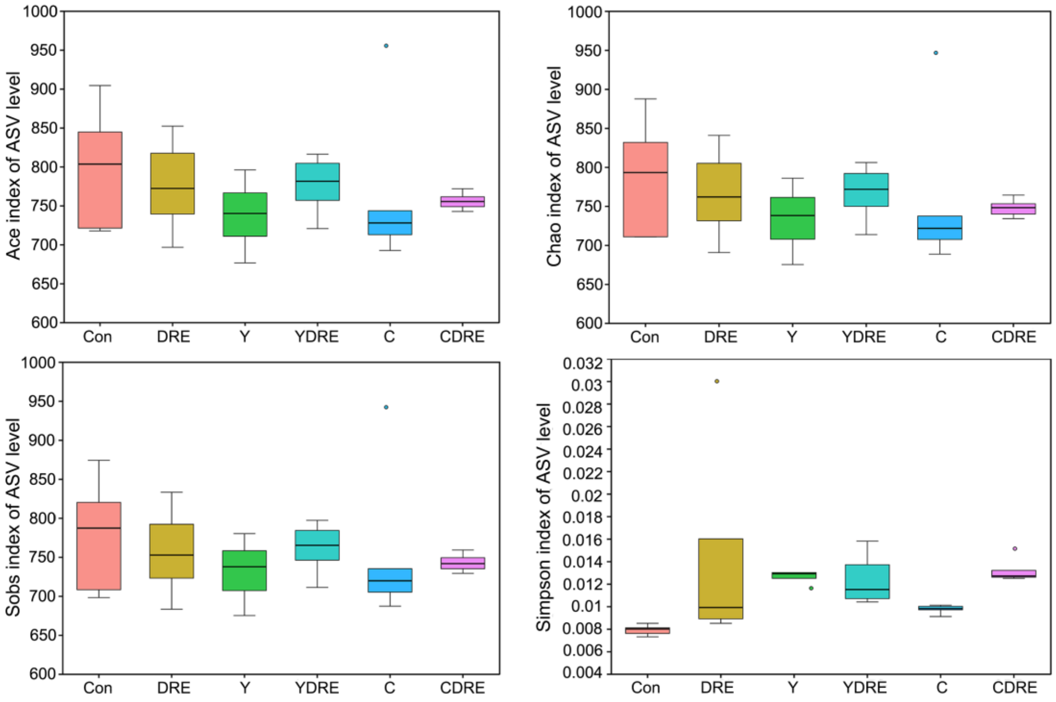

Ace index of ASV level
Chao index of ASV level
Sobs index of ASV level
Simpson index of ASV level
Con
DRE
Y
YDRE
C
CDRE

Fig. S10

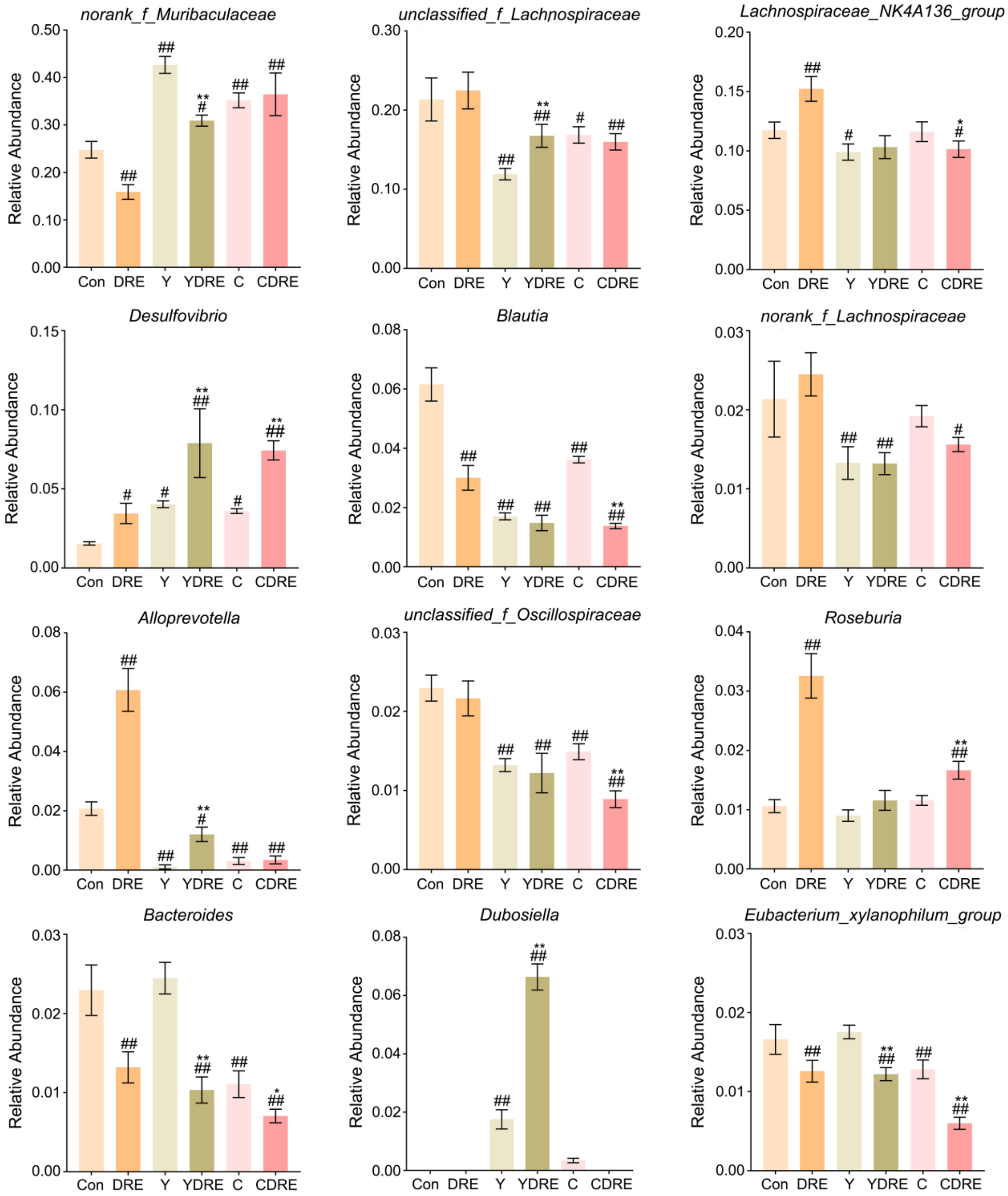

norank_f_Muribaculaceae
unclassified_f_Lachnospiraceae
Lachnospiraceae_NK4A136_group
Desulfovibrio
Blautia
norank_f_Lachnospiraceae
Alloprevotella
unclassified_f_Oscillospiraceae
Roseburia
Bacteroides
Dubosiella
Eubacterium_xylanophilum_group
Relative Abundance
Con DRE Y YDRE C CDRE

Fig. S11

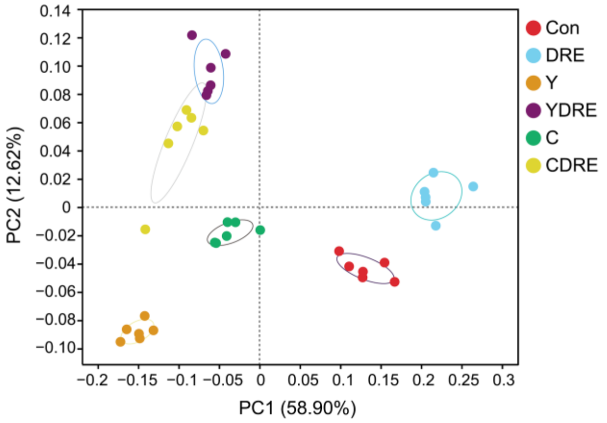

0.14
0.12
0.10
0.08
0.06
0.04
0.02
0
−0.02
−0.04
−0.06
−0.08
−0.10
PC2 (12.62%)
−0.2
−0.15
−0.1
−0.05
0
0.05
0.1
0.15
0.2
0.25
0.3
PC1 (58.90%)
Con
DRE
Y
YDRE
C
CDRE

Table S1

Tissue	Biomarker	Con	DRE	Y	YDRE	C	CDRE
**Small intestine**	IL-6 (pg·mL^{-1})	84.00±6.56	70.67±3.21	185.00±9.85$^{\#\#}$	145.00±8.89$^{\#\#,**}$	124.33±5.13$^{\#\#}$	95.00±7.00*
	IL-10 (pg·mL^{-1})	64.67±4.51	58.33±5.69	37.00±4.36$^{\#\#}$	58.67±3.51*	50.67±5.03$^{\#}$	82.00±5.57**
	TNF-α (pg·mL^{-1})	43.00±5.20	39.00±3.61	80.33±5.03$^{\#\#}$	66±4.58$^{\#\#,*}$	57.33±3.51$^{\#}$	48.00±3.00
	IFN-γ (pg·mL^{-1})	80.00±3.61	65.67±4.04	132.33±4.93$^{\#\#}$	106.67±6.51$^{\#\#,**}$	86.33±7.77	70.00±4.58$^{\#,*}$
**Colon**	IL-6 (pg·mL^{-1})	121.00±10.41	105.33±4.51	291.67±12.58$^{\#\#}$	190.00±9.17$^{\#\#,**}$	181.67±7.64$^{\#\#}$	160.00±8.00$^{\#,*}$
	IL-10 (pg·mL^{-1})	103.67±5.51	115.67±8.96	73.00±6.24$^{\#\#}$	92±7.21*	120.00±8.54$^{\#}$	128.33±7.64$^{\#}$
	TNF-α (pg·mL^{-1})	32.33±4.04	29.00±2.65	106.01±6.56$^{\#\#}$	76.00±6.56$^{\#\#,**}$	56.02±3.61$^{\#\#}$	43.34±4.75*
	IFN-γ (pg·mL^{-1})	128.02±8.16	113.67±6.53	298.00±14.96$^{\#\#}$	181.10±9.54$^{\#\#,**}$	153.32±7.64$^{\#}$	142.33±7.09

Note: The data are represented as means ± SD. # indicates a significant difference compared with the control group (Con) ($^{\#}P < 0.05$, $^{\#\#}P < 0.001$). * indicates a significant difference between the Y and YDRE groups or between the C and CDRE groups ($^{*}P < 0.05$, $^{**}P < 0.001$).

Table S2

Tissue	Biomarker	Con	DRE	Y	YDRE	C	CDRE
**Small intestine**	CAT (U·mg·prot^{-1})	80.67±3.06	72.00±3.46	15.83±2.25$^{\#\#}$	28.67±2.52$^{\#\#,**}$	58.66±4.04$^{\#\#}$	63.67±3.55$^{\#\#}$
	SOD (mg·prot·mL^{-1})	21.03±1.74	19.00±0.50	3.02±1.03$^{\#\#}$	7.05±1.04$^{\#\#,**}$	13.33±1.52$^{\#\#}$	17.02±1.90$^{\#}$
	GSH-Px (μmol·min^{-1}·g^{-1})	30.00±2.65	25.77±1.66	4.80±0.72$^{\#\#}$	10.67±1.15$^{\#\#,**}$	25.33±2.08$^{\#}$	22.03±0.95$^{\#\#,**}$
	MDA (nmol·mg·prot^{-1})	0.34±0.04	0.32±0.04	1.40±0.12$^{\#\#}$	1.13±0.06$^{\#\#,*}$	0.85±0.05$^{\#\#}$	0.67±0.06$^{\#\#,*}$
	The ratio of GSH/GSSG	0.97±0.03	1.16±0.07$^{\#}$	0.58±0.06$^{\#\#}$	0.80±0.06$^{\#,*}$	0.85±0.08	1.03±0.05*
	Levels of ROS	0.88±0.07	0.96±0.08	2.10±0.12$^{\#\#}$	1.40±0.04$^{\#\#,**}$	1.23±0.06$^{\#\#}$	1.03±0.05*
**Colon**	CAT (U·mg·prot^{-1})	104.67±6.11	95.00±4.58	35.02±3.04$^{\#\#}$	68.67±3.21$^{\#\#,**}$	66.67±4.16$^{\#\#}$	80.67±2.52$^{\#\#,**}$
	SOD (mg·prot·mL^{-1})	31.67±2.89	26.67±2.08	8.33±1.53$^{\#\#}$	21.67±1.53$^{\#\#,**}$	22.67±2.52$^{\#\#}$	25.00±2.10$^{\#}$
	GSH-Px (μmol·min^{-1}·g^{-1})	41.00±3.61	42.74±3.91	10.00±1.00$^{\#\#}$	22.00±2.20$^{\#\#,**}$	32.67±2.52$^{\#}$	29.66±3.31$^{\#}$
	MDA (nmol·mg·prot^{-1})	0.62±0.12	0.62±0.13	1.78±0.11$^{\#\#}$	1.17±0.15$^{\#\#,**}$	0.90±0.06$^{\#}$	0.84±0.05$^{\#}$
	The ratio of GSH/GSSG	1.25±0.07	1.17±0.06	0.67±0.04$^{\#\#}$	0.98±0.05$^{\#,**}$	1.07±0.07$^{\#}$	0.93±0.08$^{\#\#}$
	Levels of ROS	1.13±0.09	0.94±0.05	1.87±0.10$^{\#\#}$	1.30±0.10**	1.37±0.07	1.44±0.13$^{\#}$
**Liver**	CAT (U·mg·prot^{-1})	64.00±2.65	60.00±4.36	42.02±3.00$^{\#\#}$	48.32±2.03$^{\#\#,*}$	36.00±1.73$^{\#\#}$	44.33±2.52$^{\#\#,*}$
	SOD (mg·prot·mL^{-1})	25.00±1.73	23.33±2.52	15.04±1.50$^{\#\#}$	19.33±1.15$^{\#\#,*}$	18.67±2.08$^{\#}$	22.00±2.07
	GSH-Px (μmol·min^{-1}·g^{-1})	52.67±3.01	54.67±3.51	33.00±1.73$^{\#\#}$	38.00±2.00$^{\#\#,*}$	23.36±2.52$^{\#\#}$	43.67±2.57$^{\#,**}$

MDA ($nmol \cdot mg \cdot prot^{-1}$)	1.57±0.08	1.20±0.10$^{\#\#}$	3.17±0.15$^{\#\#}$	2.33±0.06$^{\#\#,**}$	4.50±0.20$^{\#\#}$	3.14±0.20$^{\#\#,**}$
The ratio of GSH/GSSG	1.14±0.09	1.17±0.10	0.54±0.04$^{\#\#}$	0.80±0.08$^{\#\#,**}$	0.59±0.06$^{\#\#}$	0.86±0.05$^{\#\#,**}$
Levels of ROS	1.07±0.06	0.90±0.11	2.20±0.12$^{\#\#}$	1.77±0.15$^{\#\#,*}$	2.40±0.15$^{\#\#}$	1.6±0.12$^{\#\#,**}$

Note: The data are represented as means ± SD. # indicates a significant difference compared with the control group (Con) ($^{\#}P < 0.05$, $^{\#\#}P < 0.001$). * indicates a significant difference between the Y and YDRE groups or between the C and CDRE groups ($^{*}P < 0.05$, $^{**}P < 0.001$).

Table S3

Biomarker	Con	DRE	Y	YDRE	C	CDRE
ALT (U/L)	55.00±3.00	58.34±6.03	103.00±7.00$^{\#\#}$	79.45±8.02$^{\#\#,*}$	85.26±5.51$^{\#\#}$	70.41±5.04$^{\#,*}$
AST (U/L)	82.00±7.55	74.10±6.00	135.04±13.00$^{\#\#}$	105.67±10.01$^{\#,*}$	145.00±9.00$^{\#\#}$	113.67±8.02$^{\#,*}$

Note: The data are represented as means ± SD. # indicates a significant difference compared with the control group (Con) ($^{\#}P < 0.05$, $^{\#\#}P < 0.001$). * indicates a significant difference between the Y and YDRE groups or between the C and CDRE groups ($^{*}P < 0.05$, $^{**}P < 0.001$).

Table S4

Biomarkers	Small intestine						Colon					
	Con	DRE	Y	YDRE	C	CDRE	Con	DRE	Y	YDRE	C	CDRE
IL-6	0.33	0	2.81	1.83	1.32	0.6	0.27	0	2.99	1.36	1.23	0.88
IL-10	1.06	1.50	2.95	1.47	2.02	0	1.70	1.10	3.22	2.28	0.89	0.47
TNF-α	0.26	0.00	2.74	1.79	1.21	0.60	0.11	0	2.75	1.67	0.96	0.51
IFN-γ	0.60	0.00	2.80	1.70	0.86	0.18	0.23	0.00	2.91	1.06	0.63	0.45
CAT	0.41	0.77	3.12	2.57	1.33	1.12	0.45	0.87	3.44	2.00	2.08	1.48
SOD	0.41	0.71	3.08	2.49	1.55	1.01	0.71	1.36	3.77	2.02	1.89	1.58
GSH-Px	0.50	0.96	3.20	2.57	1.00	1.36	0.71	0.56	3.34	2.32	1.42	1.67
MDA	0.06	0.00	2.65	1.99	1.3	0.85	0	0.02	2.76	1.30	0.66	0.53
The ratio of GSH/GSSG	1.27	0.27	3.23	2.10	1.88	0.94	0.54	0.92	3.46	1.87	1.46	2.12
Levels of ROS	0	0.18	2.85	1.21	0.82	0.35	0.64	0.00	3.04	1.18	1.41	1.64
Pathological score	0.26	0	2.89	1.58	1.32	0.79	0.2	0.0	2.86	1.53	1.06	0.59
R_{IB}	0.43	0.35	25.59	11.38	5.13	1.58	0.57	0.53	29.30	8.38	4.65	3.64

Biomarkers	Liver					
	Con	DRE	Y	YDRE	C	CDRE
ALT	0.00	0.19	2.72	1.38	1.73	0.87
AST	0.29	0.00	2.22	1.15	2.58	1.44
CAT	0.00	0.20	1.93	1.35	2.50	1.70
SOD	0.54	0.02	2.76	1.80	1.95	1.21
GSH-Px	0.5	0.33	2.22	1.78	3.07	1.29
MDA	0.32	0	1.72	0.99	2.89	1.69
The ratio of GSH/GSSG	0.12	0.00	2.44	1.41	2.23	1.17
Levels of ROS	0.30	0.00	2.29	1.53	2.64	1.23
Pathological score	0.07	0.00	2.32	1.05	2.40	1.42
R_{IB}	0.14	0.00	12.68	4.63	14.43	4.29
