# Supplementary figures and images for: Protective Effect of Dictyophora rubrovolvata Extract on Intestinal and Liver Tissue Toxicity Induced by Metformin Disinfection Byproducts
Source: Toxics. 2025 Apr 16;13(4):310. doi: 10.3390/toxics13040310 (PMC12030868; doi:10.3390/toxics13040310)

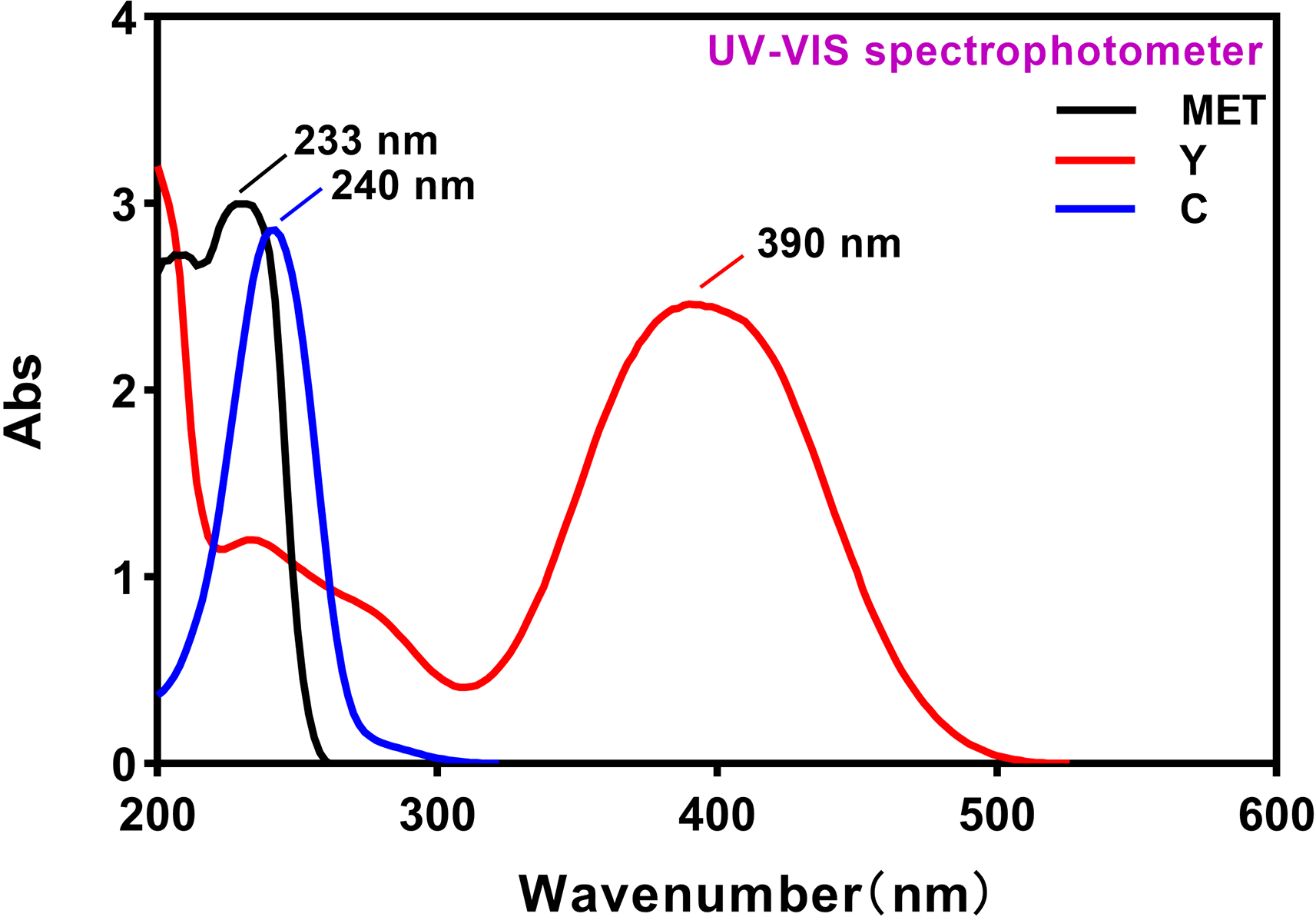

Supplement: Supplementary file 1 [file toxics-13-00310-s001.zip › Supplementary Fig 1.tif]

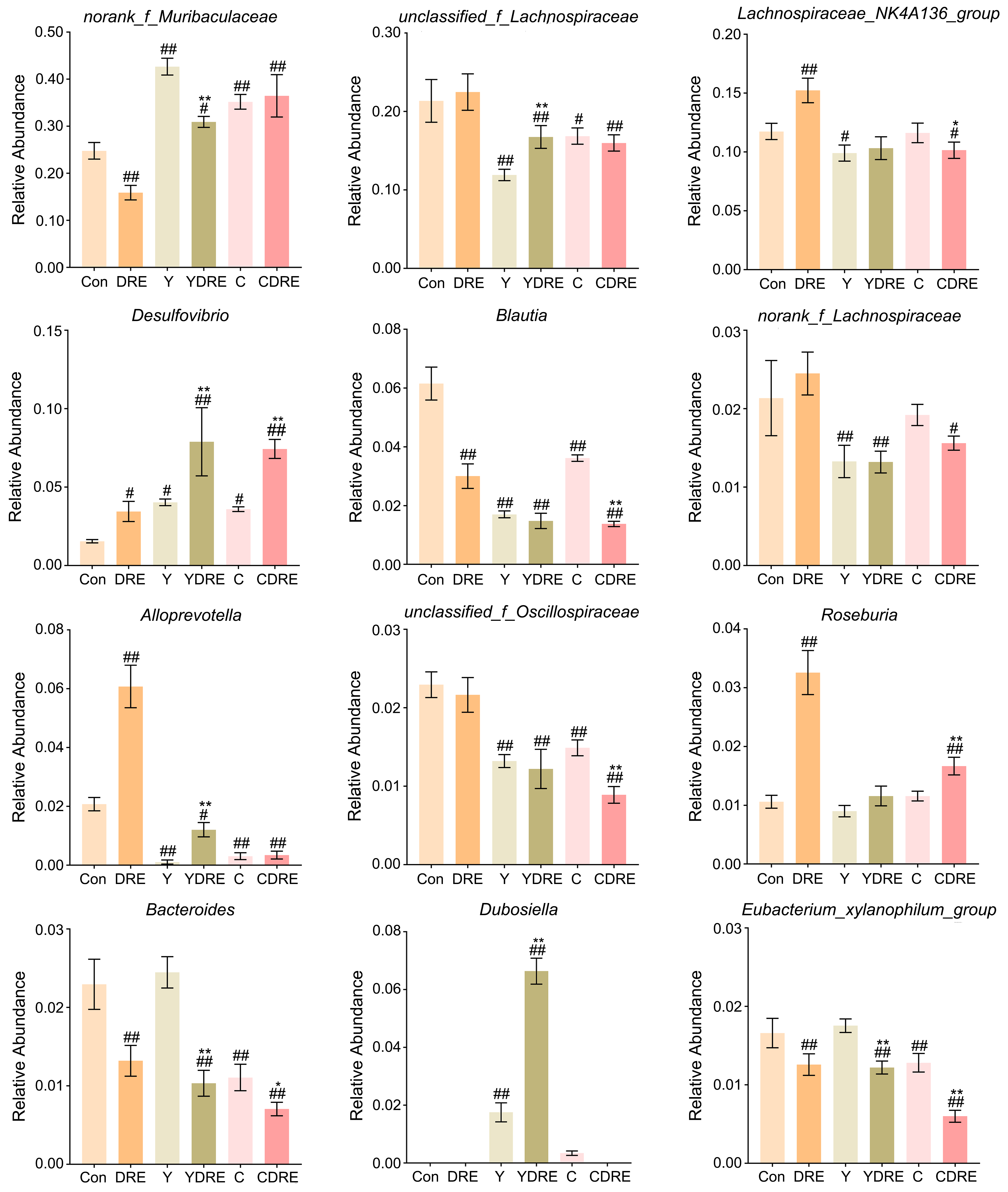

Supplement: Supplementary file 1 [file toxics-13-00310-s001.zip › Supplementary Fig 10.tif]

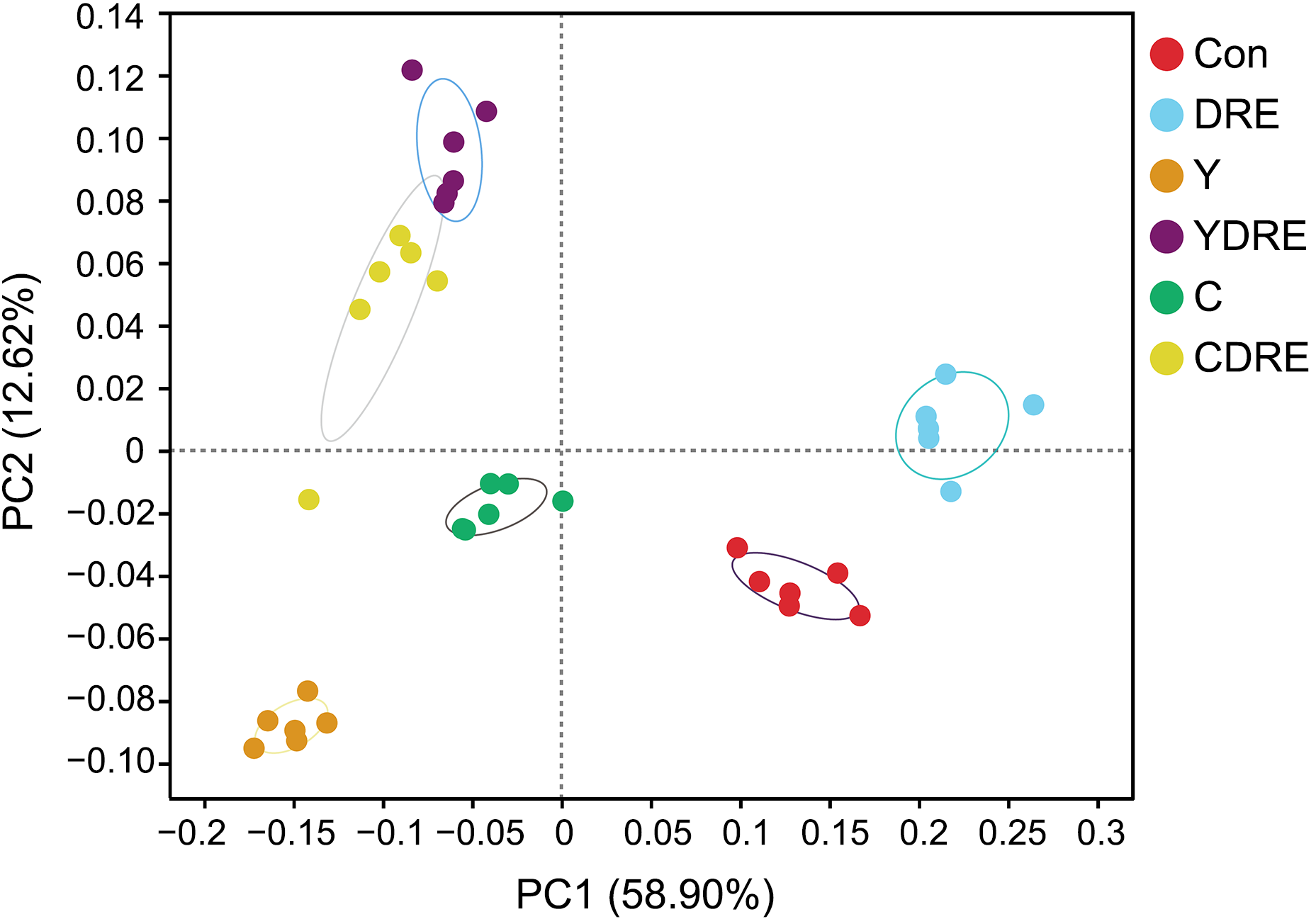

Supplement: Supplementary file 1 [file toxics-13-00310-s001.zip › Supplementary Fig 11.tif]

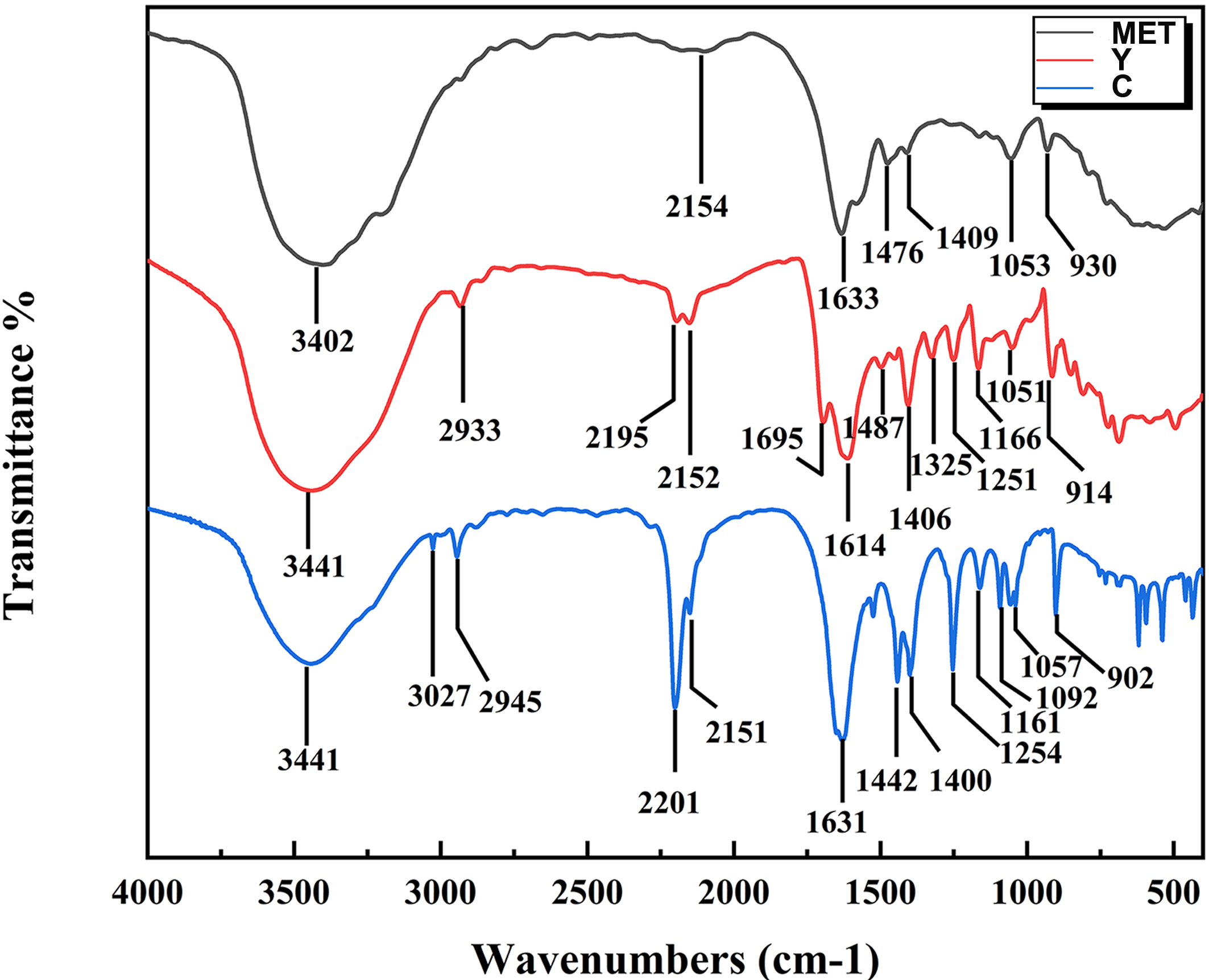

Supplement: Supplementary file 1 [file toxics-13-00310-s001.zip › Supplementary Fig 2.tif]

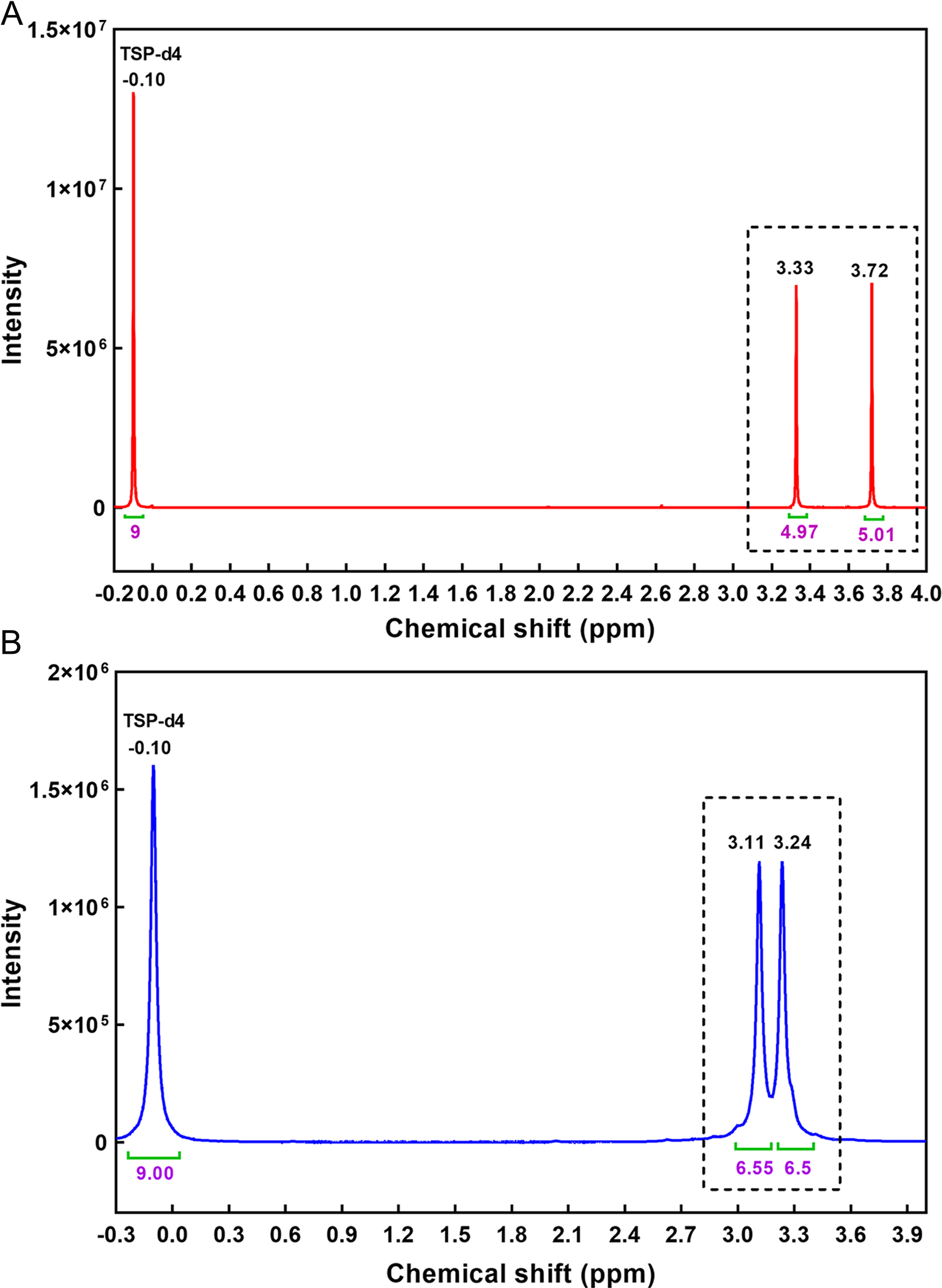

Supplement: Supplementary file 1 [file toxics-13-00310-s001.zip › Supplementary Fig 3.tif]

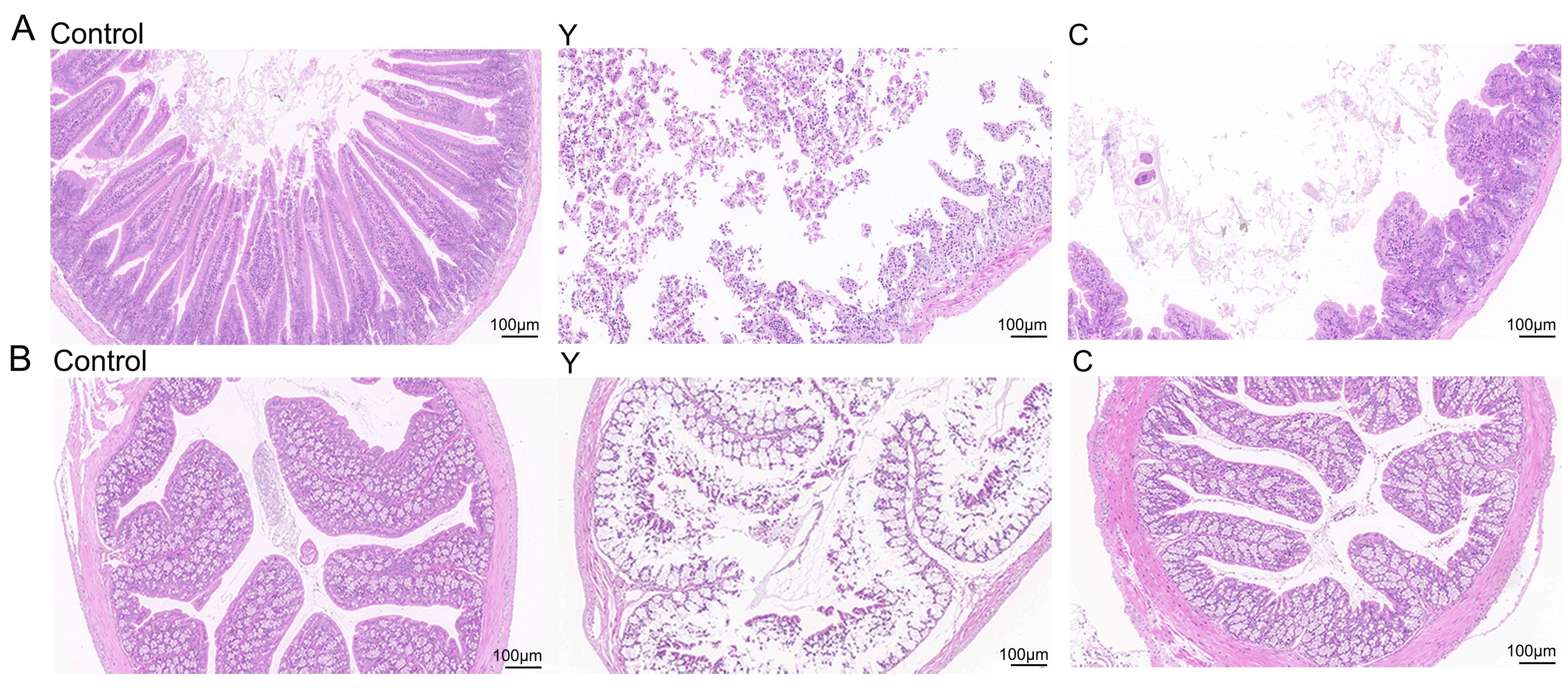

Supplement: Supplementary file 1 [file toxics-13-00310-s001.zip › Supplementary Fig 4.tif]

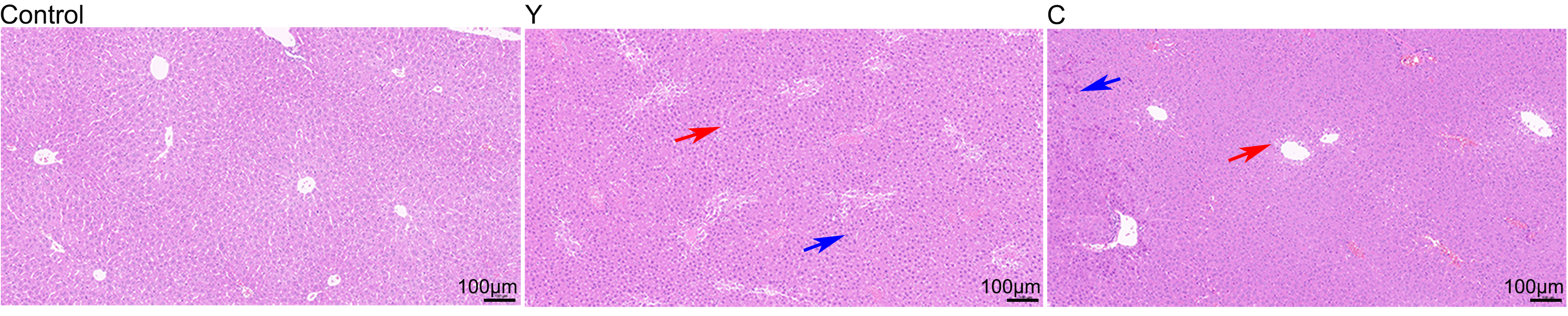

Supplement: Supplementary file 1 [file toxics-13-00310-s001.zip › Supplementary Fig 5.tif]

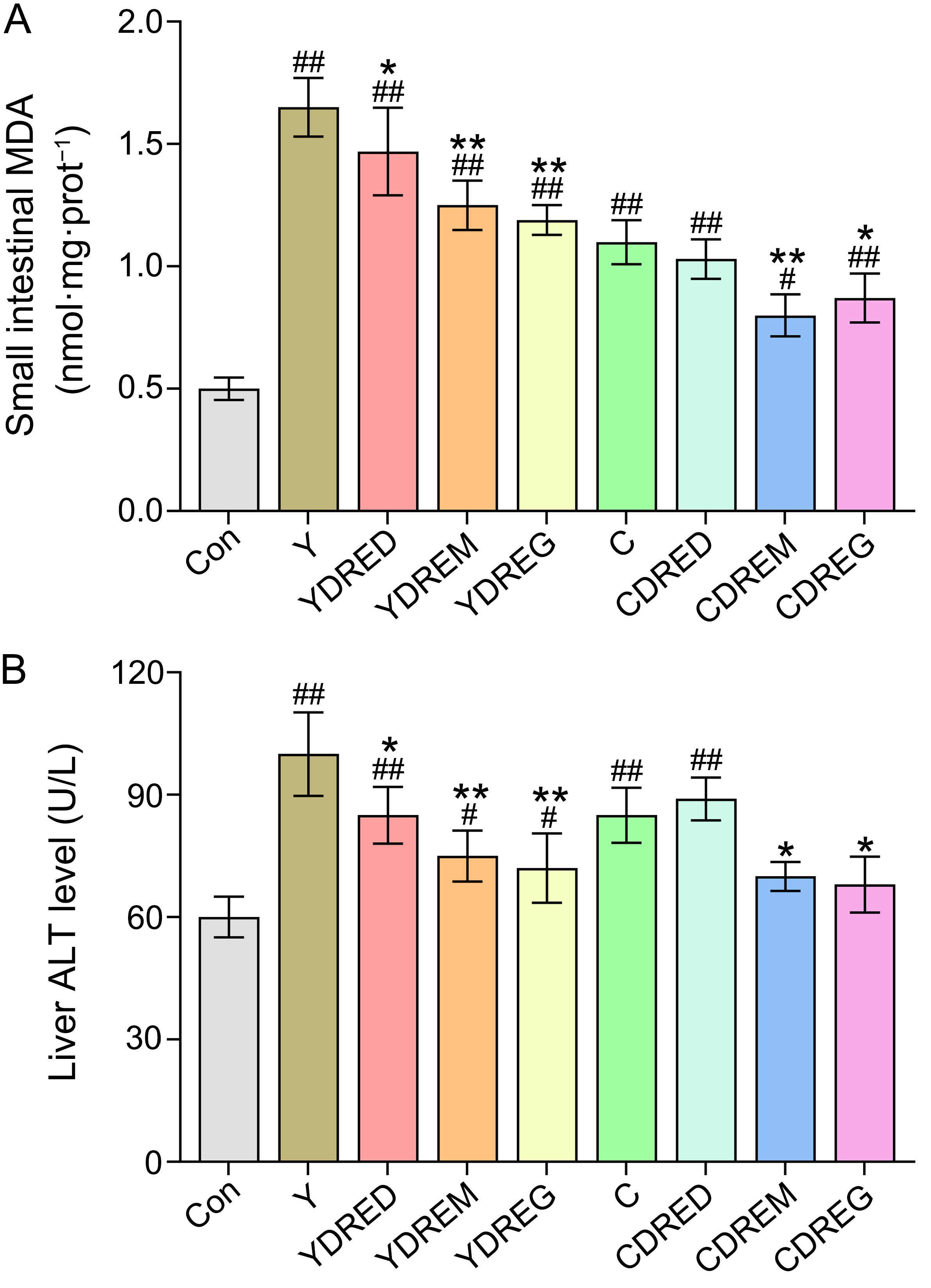

Supplement: Supplementary file 1 [file toxics-13-00310-s001.zip › Supplementary Fig 6.tif]

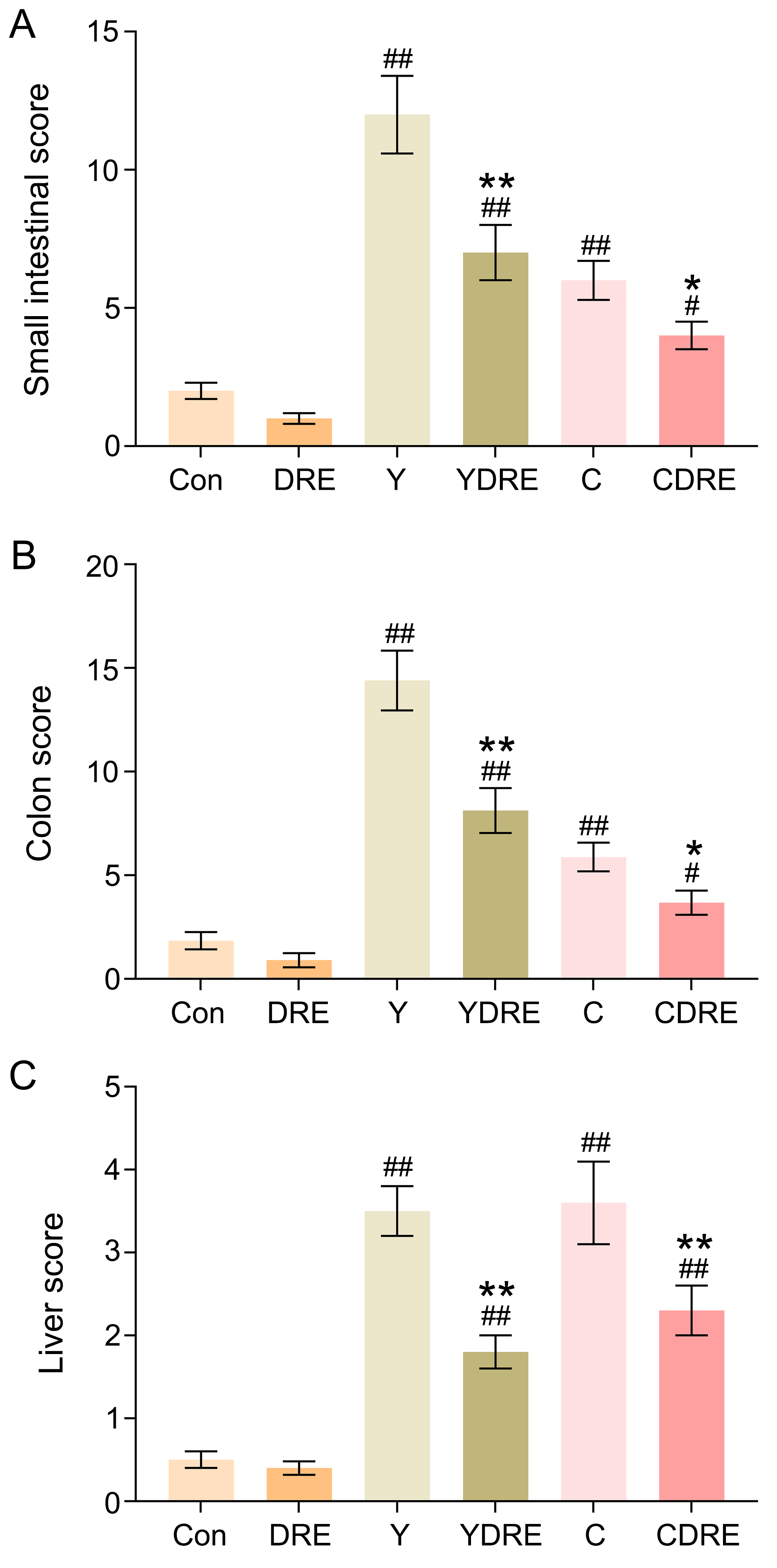

Supplement: Supplementary file 1 [file toxics-13-00310-s001.zip › Supplementary Fig 7.tif]

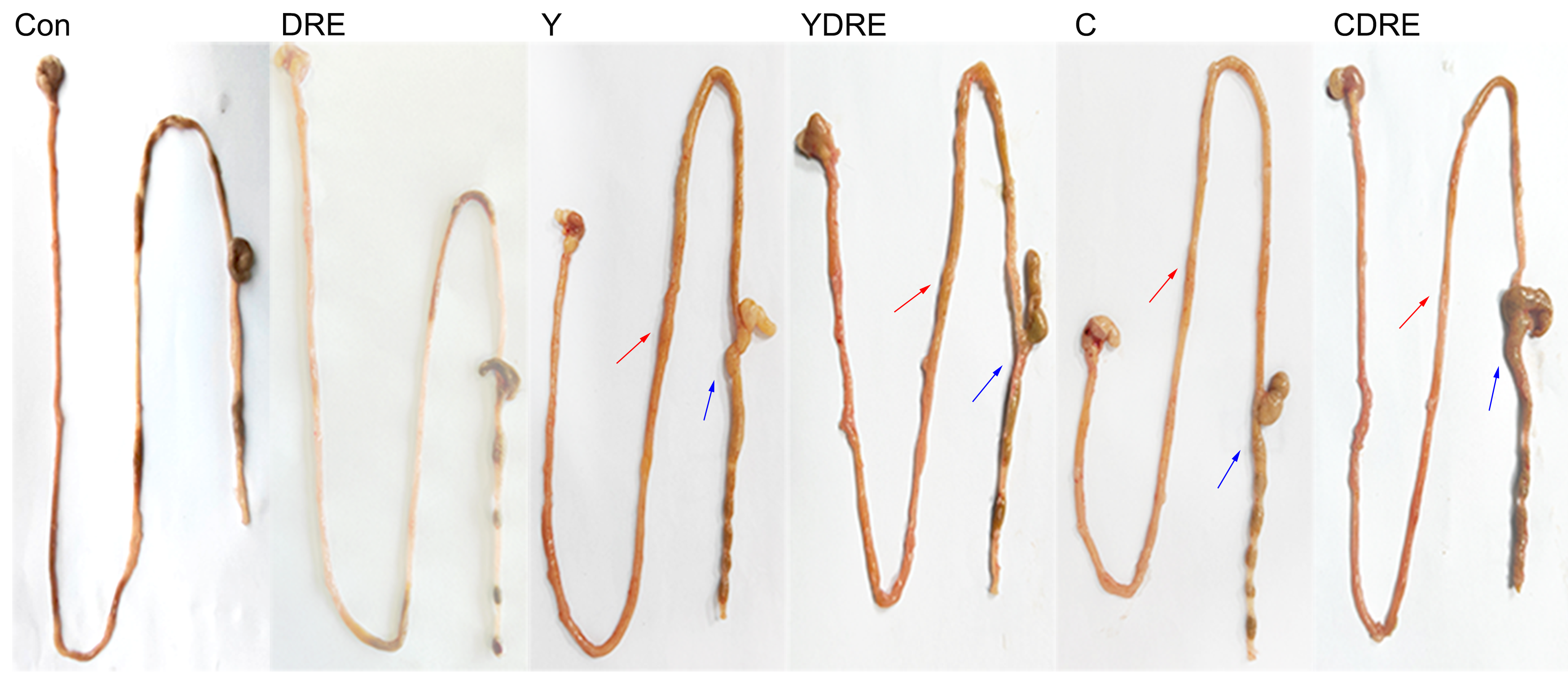

Supplement: Supplementary file 1 [file toxics-13-00310-s001.zip › Supplementary Fig 8.tif]

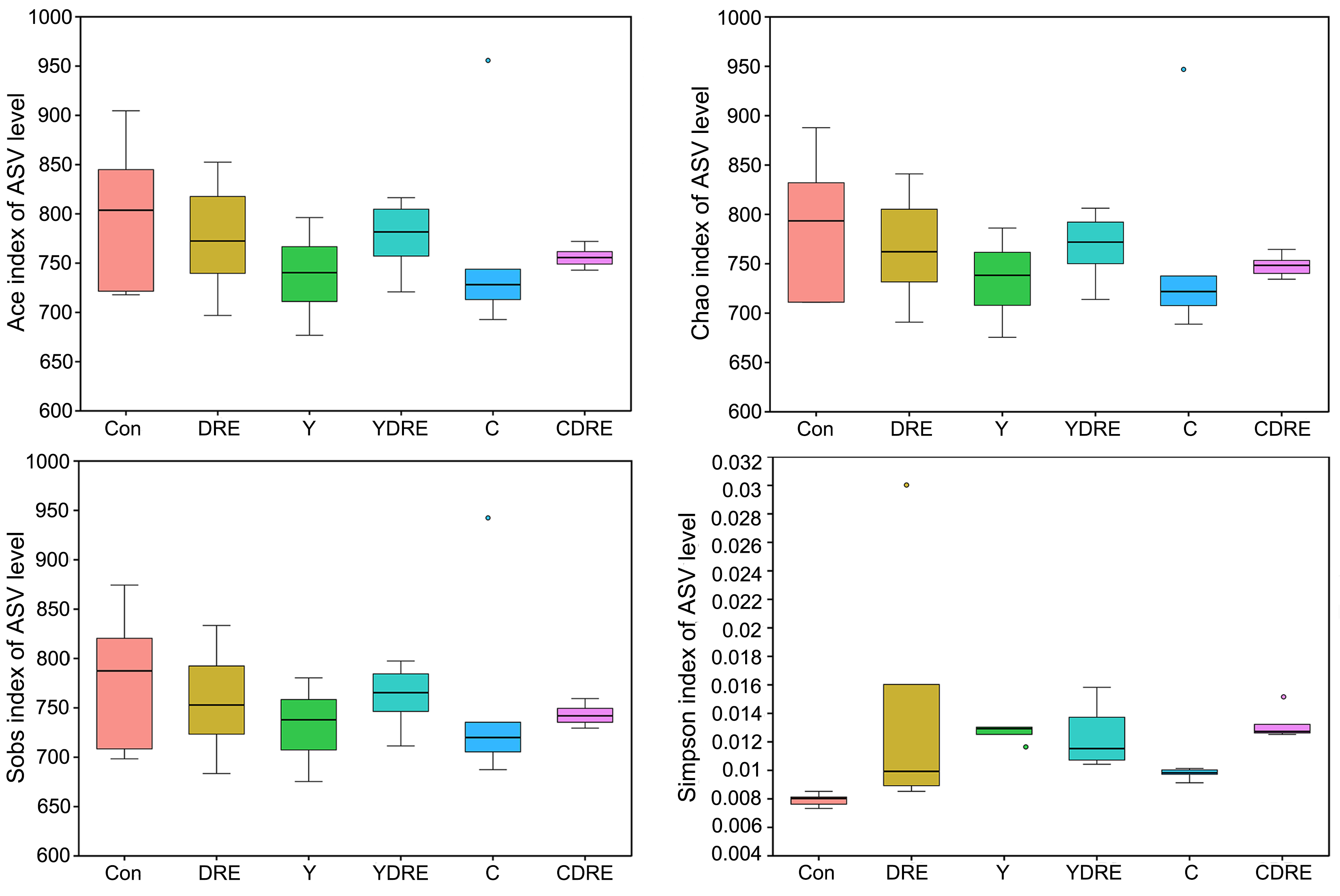

Supplement: Supplementary file 1 [file toxics-13-00310-s001.zip › Supplementary Fig 9.tif]
